# Benchmarking foundation models as feature extractors for weakly supervised computational pathology

**DOI:** 10.1038/s41551-025-01516-3

**Published:** 2025-10-01

**Authors:** Peter Neidlinger, Omar S. M. El Nahhas, Hannah Sophie Muti, Tim Lenz, Michael Hoffmeister, Hermann Brenner, Marko van Treeck, Rupert Langer, Bastian Dislich, Hans Michael Behrens, Christoph Röcken, Sebastian Foersch, Daniel Truhn, Antonio Marra, Oliver Lester Saldanha, Jakob Nikolas Kather

**Affiliations:** 1 Else Kroener Fresenius Center for Digital Health, Faculty of Medicine and University Hospital Carl Gustav Carus, TUD Dresden University of Technology, Dresden, Germany; 2 StratifAI GmbH, Dresden, Germany; 3 Department for Visceral, Thoracic and Vascular Surgery, University Hospital and Faculty of Medicine Carl Gustav Carus, TUD Dresden University of Technology, Dresden, Germany; 4 National Center for Tumor Diseases Dresden (NCT/UCC), a partnership between DKFZ, Faculty of Medicine and University Hospital Carl Gustav Carus, TUD Dresden University of Technology and Helmholtz-Zentrum Dresden - Rossendorf (HZDR), Dresden, Germany; 5 Division of Clinical Epidemiology and Aging Research, German Cancer Research Center (DKFZ), Heidelberg, Germany; 6 Division of Preventive Oncology, German Cancer Research Center (DKFZ) and National Center for Tumor Diseases (NCT), Heidelberg, Germany; 7 German Cancer Consortium (DKTK), German Cancer Research Center (DKFZ), Heidelberg, Germany; 8 Institute of Pathology and Molecular Pathology, Kepler University Hospital, Johannes Kepler University Linz, Linz, Austria; 9 Institute of Tissue Medicine and Pathology, University of Bern, Bern, Switzerland; 10 Department of Pathology, University Hospital Schleswig-Holstein, Kiel, Germany; 11 Institute of Pathology, University Medical Center Mainz, Mainz, Germany; 12 Department of Diagnostic and Interventional Radiology, University Hospital Aachen, Aachen, Germany; 13 Division of New Drugs and Early Drug Development, European Institute of Oncology IRCCS, Milan, Italy; 14 Department of Medicine I, Faculty of Medicine and University Hospital Carl Gustav Carus, TUD Dresden University of Technology, Dresden, Germany; 15 Medical Oncology, National Center for Tumor Diseases (NCT), University Hospital Heidelberg, Heidelberg, Germany

**Keywords:** Cancer imaging, Tumour biomarkers

## Abstract

Numerous pathology foundation models have been developed to extract clinically relevant information. There is currently limited literature independently evaluating these foundation models on external cohorts and clinically relevant tasks to uncover adjustments for future improvements. Here we benchmark 19 histopathology foundation models on 13 patient cohorts with 6,818 patients and 9,528 slides from lung, colorectal, gastric and breast cancers. The models were evaluated on weakly supervised tasks related to biomarkers, morphological properties and prognostic outcomes. We show that a vision-language foundation model, CONCH, yielded the highest overall performance when compared with vision-only foundation models, with Virchow2 as close second, although its superior performance was less pronounced in low-data scenarios and low-prevalence tasks. The experiments reveal that foundation models trained on distinct cohorts learn complementary features to predict the same label, and can be fused to outperform the current state of the art. An ensemble combining CONCH and Virchow2 predictions outperformed individual models in 55% of tasks, leveraging their complementary strengths in classification scenarios. Moreover, our findings suggest that data diversity outweighs data volume for foundation models.

## Abstract (web summary)

A comprehensive benchmarking of 19 histopathology foundation models finds that a vision-language foundation model outperforms vision-only models.

## Main

Artificial intelligence has revolutionized digital pathology by enabling biomarker prediction from cancer tissues using high-resolution whole-slide images (WSIs)^1–6^. Moreover, these algorithms can substantially enhance diagnostic accuracy, efficiency and consistency, reducing the subjectivity associated with human interpretation^7,8^. In particular, deep learning can perform tasks such as disease grading, cancer subclassification or prognostic prediction^9–11^.

Recently, foundation models, which are trained on large-scale datasets, have been introduced to digital pathology^12,13^. These models use self-supervised learning (SSL) techniques to learn meaningful representations of histology tissue, which are crucial for clinical pathology tasks. SSL techniques such as contrastive learning^14,15^ and masked image modelling^16^ have shown improved performance, robustness and higher transferability compared with fully supervised learning. Another advantage lies in its ability to learn from vast amounts of unlabelled data, thereby considerably reducing the need for manual annotation^17^. The practical application of foundation models involves WSI tessellation into small, non-overlapping patches, after which image feature extraction is performed. These extracted features serve as inputs for training classification or regression models, such as ViTs^18^, tailored for specific tasks, such as mutation prediction, survival analysis, disease grading or cancer classification^19^. The limited availability and variable quality of public pathology data can hinder the performance of these models when applied to real-world clinical scenarios^20^. Recent efforts have demonstrated the potential of large-scale foundation models in computational pathology. Unlike earlier models that relied heavily on datasets such as The Cancer Genome Atlas (TCGA), contemporary foundation models are now trained on much larger proprietary cohorts such as Mass-100K (100,000 WSIs)^21^, Providence (171,000 WSIs)^22^ and Memorial Sloan Kettering Cancer Center (1,488,000 WSIs)^23^.

Foundation models have enabled the rapid development of specialized, task-specific downstream models by providing a stable base architecture. These downstream models require substantially less data and computational resources since they build upon the pre-existing foundation model. While the success of foundation models is typically measured by downstream model performance, their evaluation has largely been limited to narrow benchmarks without proper external validation. This restricted testing approach risks data leakage and selective reporting of only the best-performing models. As a result, most foundation models lack systematic evaluation across a broad spectrum of clinically relevant tasks, leaving their true capabilities and limitations incompletely understood.

In this study, we put forth a comprehensive benchmarking effort for histopathology foundation models. By including multiple proprietary cohorts from multiple countries, which were never part of any foundation model training, we effectively mitigate the risk of data leakage from pretraining datasets. Our benchmarking includes 19 foundation models and 31 clinically relevant evaluation tasks, 19 of which are the prediction of cancer biomarkers, using a total of 6,818 patients and 9,528 slides. This comprehensive evaluation bridges a notable gap in digital pathology literature and will serve as an important reference point for the digital pathology community helping to select the right foundation model for a specific digital pathology task.

## Results

### Benchmark of pathology foundation models

**Fig. 1:**
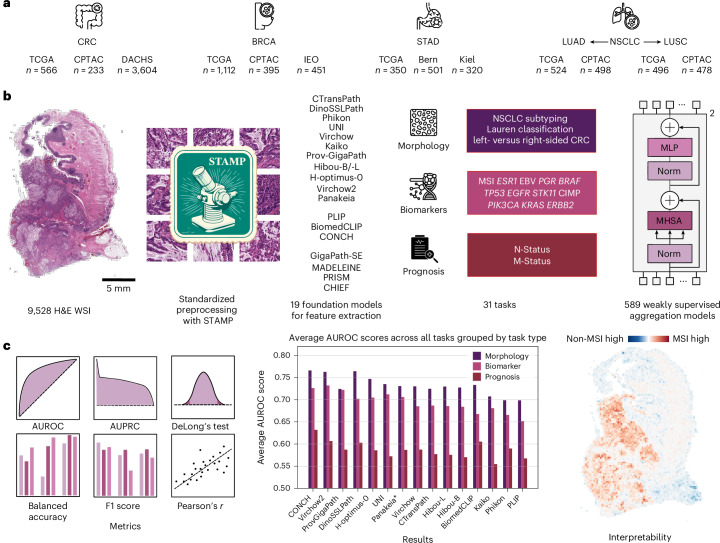
Experimental design of the study. Benchmarking of 19 histopathology foundation models using 13 cohorts and 31 tasks. **a**, Number of slides used from each of the 13 cohorts including 4 cancer types. **b**, About 9,528 haematoxylin and eosin (H&E) stained WSIs were preprocessed using the standardized STAMP^19^ pipeline. Feature extraction from the processed tiles was performed using 19 foundation models analysed in this study. The TCGA features were utilized for fivefold cross-validation with downstream transformer models on 31 classification tasks using STAMP. All models were subsequently applied to external features from CPTAC, Bern, Kiel, DACHS and IEO. The transformer architecture schematic shows layer normalization (Norm) and multi-headed self-attention (MSHA), followed by a MLP. **c**, All experiments were analysed using AUROCs, supplemented by AUPRC, Pearson’s correlation coefficient, DeLong’s test, balanced accuracy and F1 score. CONCH achieves the highest average AUROC across all tasks, followed by Virchow2, Prov-GigaPath and DinoSSLPath. The star indicates that Panakeia was tested on all tasks despite being specifically designed for BRCA and CRC. Attention heatmaps were generated for some slides to interpret differences between foundation models. Source data

We benchmarked the performance of 19 foundation models and 14 ensembles derived from these models, trained as vision-language or vision-only, on 31 weakly supervised downstream prediction tasks related to morphology (*n* = 5), biomarkers (*n* = 19) and prognostication (*n* = 7) (Fig. 1).

**Fig. 2:**
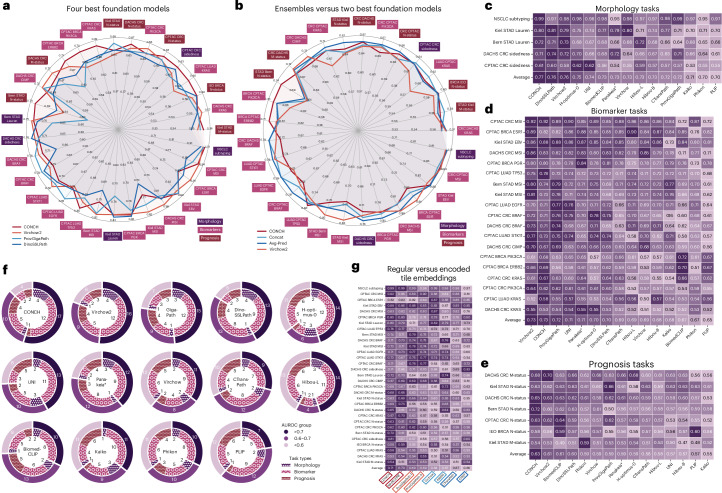
Performance of 19 pathology foundation models on 31 weakly supervised prediction tasks. **a**, AUROC scores of the four best foundation models, task-wise normalization. **b**, AUROC scores of the two best foundation models compared with the average prediction of the four best models (Avg-Pred) and the concatenated vectors of CONCH and Prov-GigaPath (Concat). **c**–**e**, Average AUROC scores of the five folds of each foundation model on morphology (**c**), biomarker (**d**) and prognosis (**e**) tasks. Task-wise normalization for better comparison of the foundation models. Tasks are sorted by their mean AUROC across all models, while models are sorted by their mean AUROC across all tasks. **f**, Stacked pie charts showing the number of tasks where each model achieved an average AUROC of >0.7, 0.6–0.7 or <0.6, grouped by task type. **g**, Average AUROC scores of the five folds using encoded tile embeddings from slide encoders versus the original tile embeddings. The star indicates that Panakeia was tested on all tasks despite being specifically designed for BRCA and CRC. Source data

**Extended Data Fig. 1:**
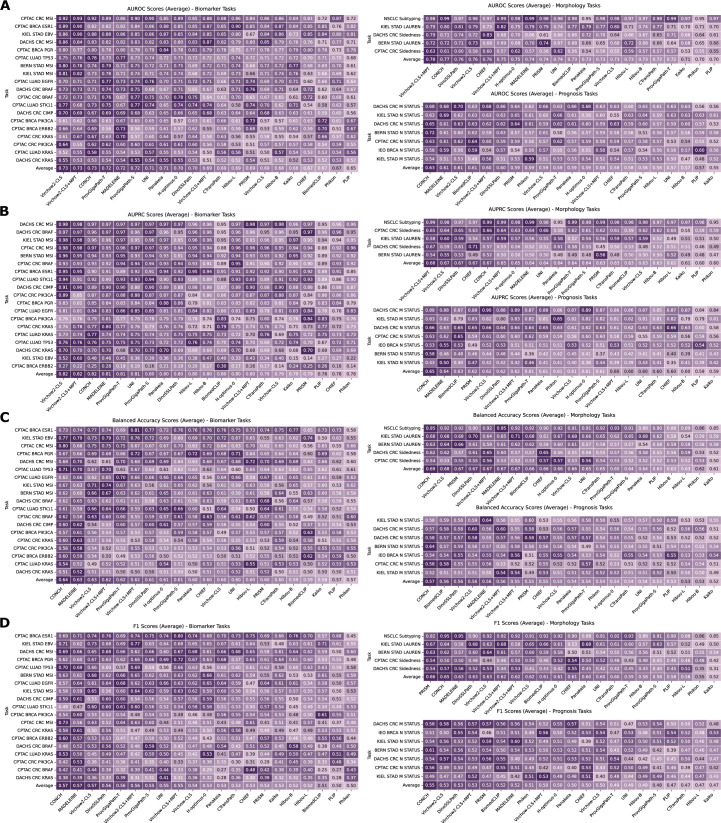
AUROCs, AUPRCs, balanced accuracy and F1-scores for all main experiments. **A-D**, Average AUROC (**A**), AUPRC (**B**), balanced accuracy (**C**) and F1 (**D**) scores of the five-folds of each foundation model on Morphology, Biomarker and Prognosis tasks. Source data

**Extended Data Fig. 2:**
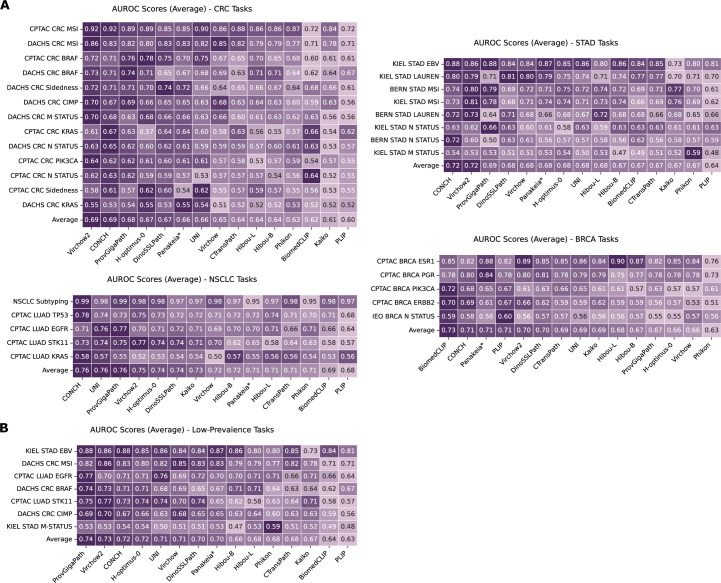
Average AUROCs sorted by cancer type and on scarce data tasks. Average AUROC scores of the five folds of each foundation model. Taskwise normalization for better comparison of the foundation models. Tasks are sorted by their mean AUROC across all models, while models are sorted by their mean AUROC across all tasks. **A**, The 31 tasks were grouped by cancer type (5 tasks for NSCLC, 5 tasks for BRCA, 8 tasks for STAD, 13 tasks for CRC). Models are sorted by average performance. **B**, Only tasks with rare positive cases (>15%) in the TCGA training cohort are shown. To avoid cancer type imbalance, these tasks are only evaluated in DACHS, Kiel and CPTAC LUAD. Source data

**Extended Data Fig. 3:**
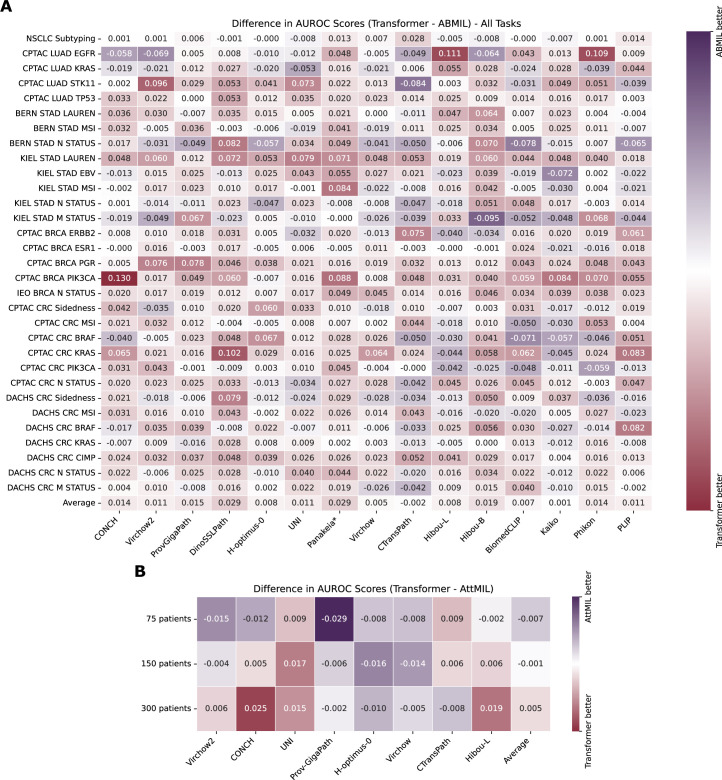
Comparison of STAMP and ABMIL. **A**, Difference in average AUROC scores between STAMP transformer-based aggregation and ABMIL across all tasks, calculated as the average over five cross-validation folds for each foundation model. Positive values indicate superior performance of STAMP. **B**, Difference in average macro-AUC scores between STAMP and ABMIL for selected foundation models under reduced downstream training dataset conditions, as shown in Extended Data Fig. 5. This compares the relative performance of both methods in low-data scenarios. Source data

For the 5 morphology-related tasks, CONCH yielded the highest mean area under the receiver operating characteristic curve (AUROC) of 0.77, followed by Virchow2 and DinoSSLPath with mean AUROCs of 0.76 (Fig. 2c). Across the 19 biomarker-related tasks, Virchow2 and CONCH achieved the highest mean AUROCs of 0.73, followed closely by Prov-GigaPath with a mean AUROC of 0.72 (Fig. 2d). Finally, in the 7 prognostic-related tasks, CONCH yielded the highest mean AUROC of 0.63, followed by Virchow2 and BiomedCLIP with mean AUROCs of 0.61 (Fig. 2e). Averaged across all 31 tasks, CONCH and Virchow2 had the highest AUROCs of 0.71, followed by Prov-GigaPath and DinoSSLPath with AUROCs of 0.69. Subsequent rankings included H-optimus-0, UNI and Panakeia (0.68), Virchow, Hibou-L and CTransPath (0.67), BiomedCLIP and Kaiko (0.66), Phikon (0.65) and PLIP (0.64). Moreover, CONCH achieved the highest average area under the precision-recall curve (AUPRC), balanced accuracy and F1 scores (Extended Data Fig. 1), with the highest average AUROC in each cancer type obtained by CONCH (stomach adenocarcinoma (STAD), non-small-cell lung cancer (NSCLC)), Virchow2 (colorectal cancer (CRC)) and BiomedCLIP (breast cancer (BRCA)) (Extended Data Fig. 2a). To further validate our findings, we compared the performance of transformer-based aggregation with the widely used attention-based multiple instance learning (ABMIL) approach^24^. Across all 31 tasks, ABMIL performed slightly worse than the transformer-based model, with an average AUROC difference of 0.01, leaving the overall model rankings largely unchanged (Extended Data Fig. 3).

For histopathology slide encoders, we retrieved the encoded tile-level embeddings to make them applicable to our MIL approach. The original tile embeddings consistently outperformed their slide-level counterparts and the performance of the encoded tile embeddings is driven by the quality of the original tile embeddings and not by the slide encoder (Fig. 2g).

**Extended Data Fig. 4:**
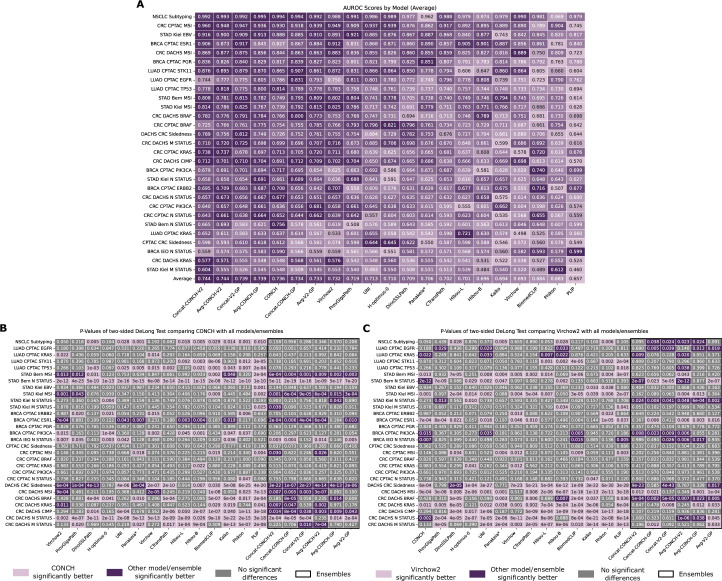
Performance Comparison of Model Ensembles and Single-Model Baselines Using DeLong’s Test. **A**, AUROC scores for each model and ensemble approach are shown, averaging predictions across five folds for individual models and five or ten folds for ensembles. Two ensembling approaches were used: taking the average prediction scores of downstream models trained on different foundation model backbones (prefix Avg) and concatenating feature vectors from different backbones to create a single downstream model (prefix Concat). The “Lauren” task was excluded as it’s not a binary classification. **B-C**, P-values from two-sided DeLong’s tests comparing CONCH (**B**) or Virchow2 (**C**) with other models and ensembles. No correction for multiple testing was applied; alpha was set to 0.05. Source data

In statistical AUROC comparisons across 29 binary classification tasks, CONCH yielded higher AUROCs, which were significantly different from other models in a substantial number of tasks: PLIP (16), Phikon and BiomedCLIP (13), Kaiko (11) and 7 tasks each for Hibou-L, H-optimus-0, CTransPath, Virchow, Panakeia, UNI and DinoSSLPath, with 5 tasks each for Prov-GigaPath and Virchow2. Conversely, few models yielded higher AUROCs than CONCH: Virchow2 (6), Prov-GigaPath (3), Panakeia and Kaiko (2) and DinoSSLPath, UNI, Virchow and Hibou-L (1). Notably, PLIP, Phikon, BiomedCLIP, H-optimus-0 and CTransPath were not significantly better than CONCH in any of the tasks (*P* < 0.05; Extended Data Fig. 4b). Among the vision-only models, Virchow2 was significantly better than all other models in between 6 and 12 tasks (*P* < 0.05; Extended Data Fig. 4c).

Together, these data show that CONCH, a vision-language model trained on 1.17 million image-caption pairs (ICPs), performs on par with Virchow2, a vision-only model trained on 3.1 million WSIs, and together outperform all other pathology foundation models in the three highlighted domains of morphology, biomarkers and prognostication-based prediction tasks and that slide encoders are ineffective in an MIL set-up.

### Performance of foundation models in scarce data settings

One of the predominant selling points of foundation models in computational pathology is the mitigation of the traditional requirement for extensive labelled datasets when analysing rare (molecular) events. Consequently, we analysed the performance of pathology foundation models across two dimensions: WSI count for foundation model training, and patient and positive case counts for downstream model training, with emphasis on low-prevalence scenarios that reflect real-world clinical applications.

**Fig. 3:**
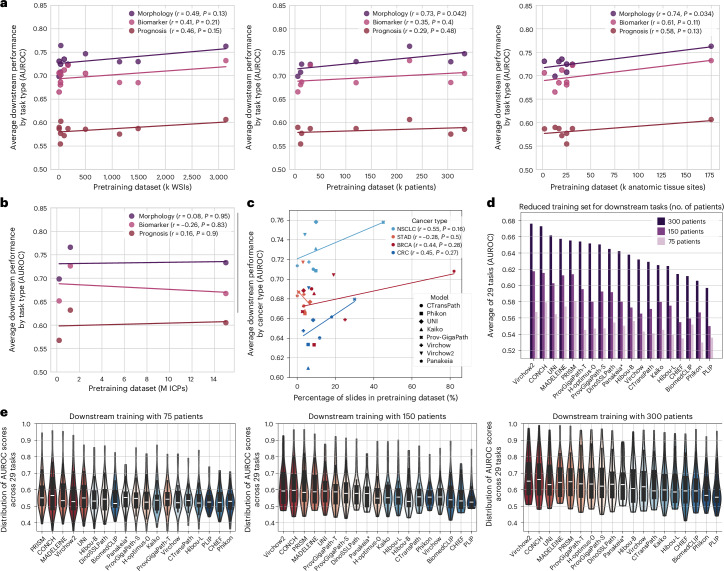
The impact of data diversity and volume on downstream weakly supervised classification performance. **a**–**c**, The impact of foundation model data diversity on downstream classification. Pearson’s correlation (two-sided) was used to assess associations between pretraining dataset characteristics and downstream performance. No adjustments were made for multiple comparisons. Correlation between the number of WSIs, patients and anatomic tissue sites in the pretraining dataset and the average AUROC for each downstream task type for all vision-only foundation models for which this data is available. Here, k denotes thousands. (**a**). Correlation between the number of ICPs (in millions, M) in the pretraining dataset and the average AUROC for each downstream task type for all vision-language foundation models (**b**). Performance of the respective cancer types correlated with the proportion of the cancer type in the pretraining dataset (**c**). All information that was available is shown (Supplementary Tables 6–8). **d**,**e**, Experiments with reduced downstream training sizes. Average AUROC scores across 29 tasks, trained with 75, 150 or 300 patients (**d**). Distribution of AUROC scores across all tasks for each model separately. Violin plots show kernel density estimates of AUROC scores, truncated at the observed range. The inner box marks the median and interquartile range (25th–75th percentiles), with whiskers extending to the most extreme values within 1.5× interquartile range (**e**). The star indicates that Panakeia was tested on all tasks despite being specifically designed for BRCA and CRC. Source data

From the foundation model perspective, positive correlations (*r* = 0.29–0.74) were observed between downstream performance and pretraining dataset size (WSIs, patients) or diversity (tissue sites) across morphology, biomarker and prognosis tasks, although most were not statistically significant. Significant correlations were found only for morphology with patient count (*r* = 0.73, *P* < 0.05) and tissue site diversity (*r* = 0.74, *P* < 0.05) (Fig. 3a). These findings suggest that these factors are important but not sole determinants, with the distribution of anatomic tissue sites (Supplementary Table 1 and Supplementary Fig. 1), architecture and dataset quality also playing critical roles. This is especially evident in vision-language models, where CONCH outperformed BiomedCLIP despite seeing far fewer ICPs (1.1 million versus 15 million) (Fig. 3b). Similarly, tissue representation in pretraining datasets showed a moderate, but not significant, correlation with performance by cancer type (Fig. 3c). Interestingly, Panakeia models showed decent performance on unrelated cancer types, with the BRCA model achieving average results in NSCLC and the CRC model performing similarly in STAD, despite no previous exposure to these tissues during training.

**Extended Data Fig. 5:**
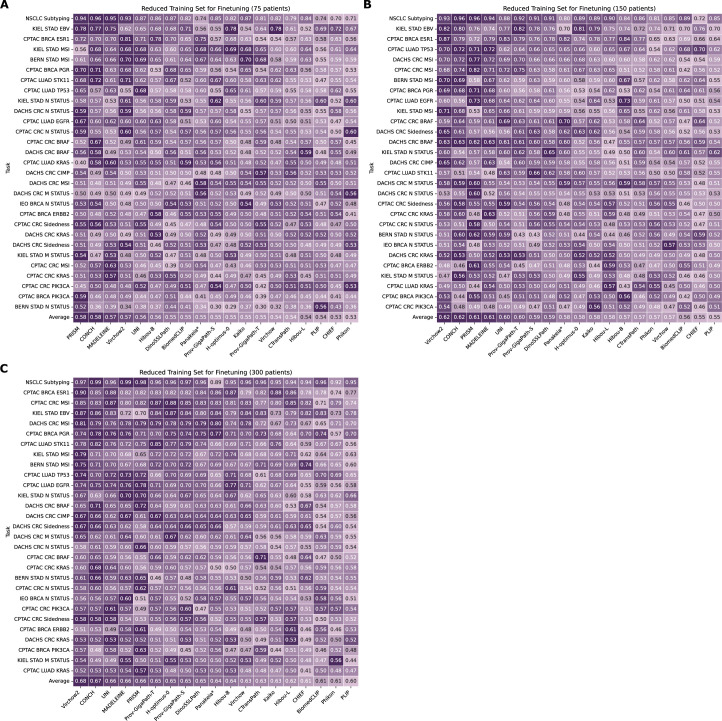
Model performance with reduced downstream training dataset. Mean AUROC across all five folds on 29 tasks for all foundation models trained with a reduced downstream dataset of 75 (**A**), 150 (**B**), or 300 patients (**C**). Patients were randomly selected from the TCGA cohorts, ensuring the ground truth was defined for all analyzed tasks. The tasks Lauren in Kiel and Bern were excluded due to insufficient patient numbers. Source data

Downstream models were trained on randomly sampled cohorts of 300, 150 and 75 patients while keeping a similar ratio of positive samples, and consequently validated on full-size external cohorts. In the largest sampled cohort (*n* = 300), Virchow2 demonstrated superior performance in 8 tasks, followed closely by PRISM with 7 tasks. With the medium-sized sampled cohort (*n* = 150), PRISM dominated by leading in 9 tasks, while Virchow2 followed with 6 tasks. The smallest sampled cohort size (*n* = 75) showed more balanced results, with CONCH leading in 5 tasks, while PRISM and Virchow2 each led in 4 tasks. Performance metrics remained relatively stable between *n* = 75 and *n* = 150 cohorts (Fig. 3d,e and Extended Data Fig. 5).

To evaluate foundation models in real-world clinical scenarios, we focused on clinically relevant tasks with rare positive cases (>15%) in the TCGA training cohort. Key low-prevalence biomarkers included *BRAF* mutation (10%), CpG island methylator phenotype (CIMP) status (13%) and microsatellite instability (MSI) status (14%) in CRC; Epstein–Barr virus (EBV) positivity (8%) and M-status (7%) in STAD; and *EGFR* mutation (11%) and *STK11* mutation (15%) in lung adenocarcinoma (LUAD). To avoid cancer type imbalance, these targets were only evaluated in DACHS, Kiel and CPTAC-LUAD. The results show that Prov-GigaPath (mean AUROC of 0.74) yields the highest performance in the highlighted low-prevalence tasks, followed by Virchow (0.73) and CONCH (0.72) (Extended Data Fig. 2b).

**Extended Data Fig. 6:**
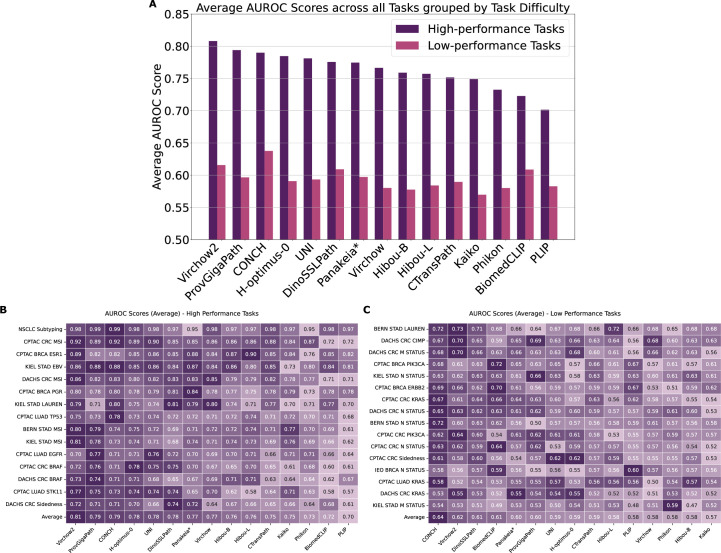
High-performance vs. low-performance tasks. **A**, Average AUROC scores across 15 high-performance and 16 low-performance tasks. Tasks were selected by including only those where at least one foundation model achieved an average AUROC over 0.75 and all others in low-performance tasks. **B-C**, The performance of each foundation model is listed. The final row presents the overall average AUROC for each model. Tasks are sorted by their mean AUROC across all models, while models are sorted by their mean AUROC across all tasks. Source data

Finally, tasks were stratified into high- and low-performance tasks by the AUROC (Extended Data Fig. 6). In high-performance tasks (>0.75), Virchow2 demonstrated superior performance in high-performance tasks, followed by Prov-GigaPath and CONCH. Conversely, in low-performance tasks (≤0.75), CONCH yielded better results.

Together, these results indicate that the patient count, tissue site diversity and their distribution are important for downstream performance, although other factors such as architecture and dataset quality also have critical roles. Moreover, the performance in downstream tasks with low-prevalence cases indicates the limitations of current foundation models for nonetheless clinically relevant biomarkers. Lastly, we show differential model efficacy based on task complexity, with Virchow2 excelling in standard classification tasks while CONCH dominates in more challenging predictive scenarios. All models show similar performance declines with reduced training sizes, underlining the weakness of current pathology foundation models in scarce data scenarios.

### Foundation models learn different tissue morphologies

**Fig. 4:**
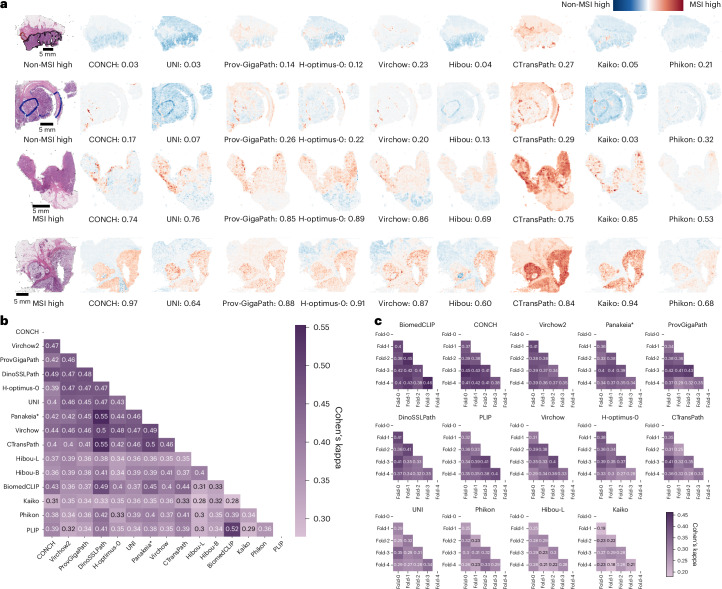
Divergence in tissue focus and predictive similarity among foundation models. **a**, Attention heatmap analysis for MSI-H classification in four different DACHS samples selected for correct predictions across selected foundation models. Thumbnails of the original WSIs and heatmaps of selected foundation models. **b**, Objective measure of similarity of prediction scores using Cohen’s kappa and majority vote across the five folds to binarize the predictions. Kappa scores of all combinations of foundation models tested in this study. **c**, Cohen’s kappa between the five folds of each foundation model. The star indicates that Panakeia was tested on all tasks despite being specifically designed for BRCA and CRC. Source data

To quantitatively measure prediction similarity across models, we calculated Cohen’s kappa^25^. For each task, labels were assigned using a majority vote across the cross-validation folds. Cohen’s kappa scores were generally moderate and varied across models. Notably, some pairs such as Panakeia and DinoSSLPath (0.55), PLIP and BiomedCLIP (0.52) and top performers such as Prov-GigaPath, CONCH, Virchow2 and DinoSSLPath showed higher agreement, whereas lower-performing models such as Hibou and Kaiko exhibited the least consensus (0.28) (Fig. 4b). Within individual model folds, BiomedCLIP and CONCH achieved the highest average kappa (0.41), followed by Virchow2, Panakeia and Prov-GigaPath (0.37), with Hibou (0.26) and Kaiko (0.24) ranking lowest, consistent with their AUROC performance (Fig. 4c).

**Extended Data Fig. 7:**
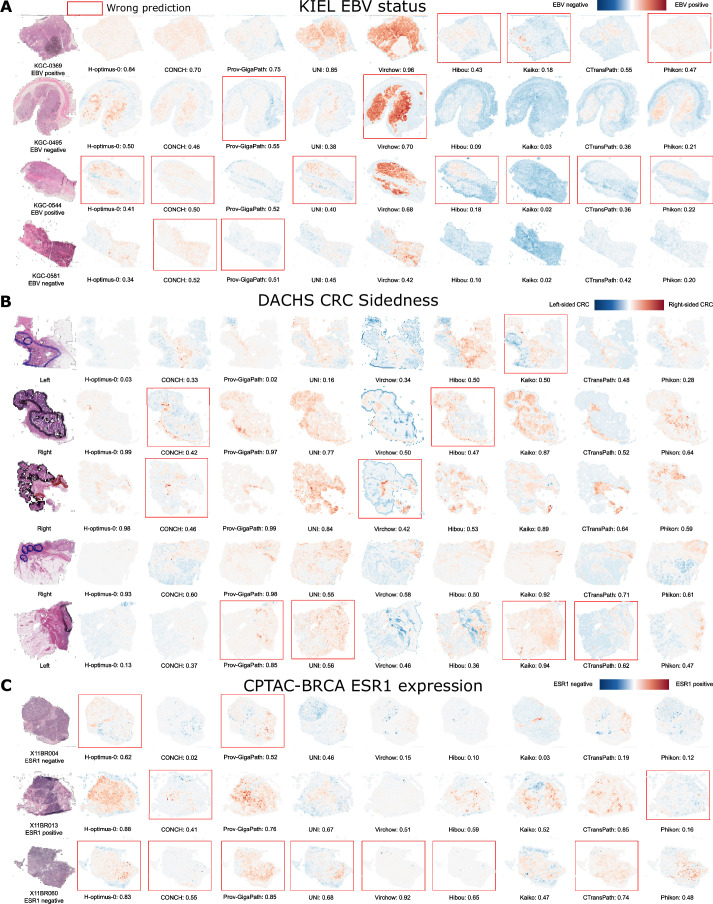
Attention heatmaps of slides with large variations in prediction scores. **A-C**, Attention Heatmap Analysis for Kiel EBV status (**A**), DACHS CRC sidedness (**B**) and CPTAC-BRCA ESR1 expression (**C**). Classification in 12 different WSIs selected for diverse prediction scores across the foundation models. Thumbnails of the original whole slide images (WSIs) and heatmaps of all foundation models are shown.

**Extended Data Fig. 8:**
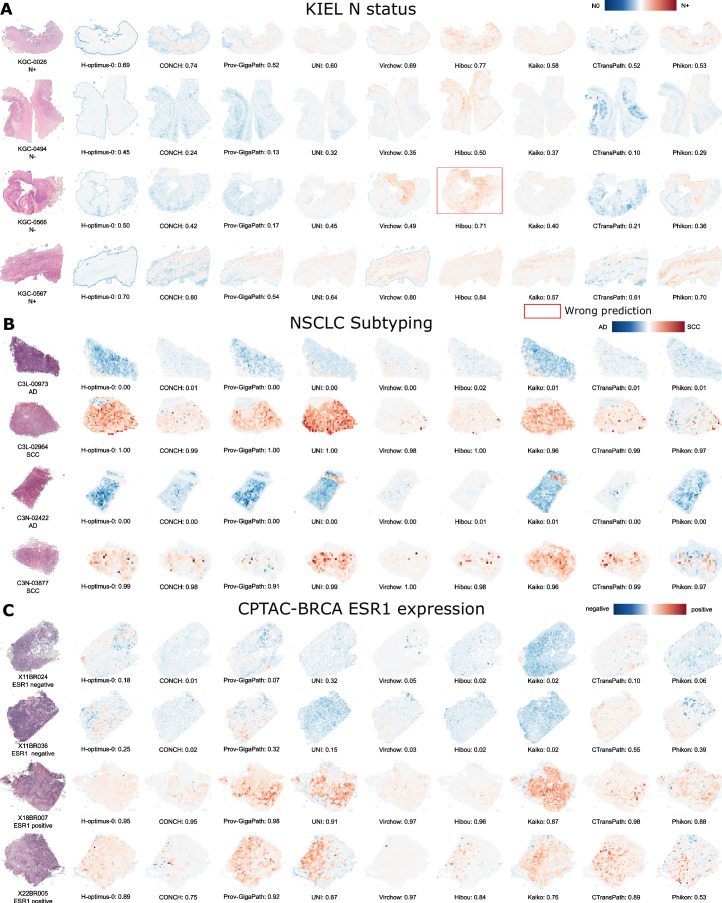
Attention heatmaps of slides that all models predicted well. **A-C**, Attention Heatmap Analysis for Kiel N status (**A**), NSCLC subtyping (**B**) and CPTAC-BRCA ESR1 expression (**C**). Classification in four different samples per cohort selected for correct predictions across almost all foundation models. Thumbnails of the original whole slide images (WSIs) and heatmaps of all foundation models are shown.

To identify the reasons behind the observed performance differences among the downstream models trained on top of the different foundation models, we investigated whether the models focus on different morphological properties for their predictions. We utilized attention heatmaps to compare model behaviour when the models (1) consistently predicted the label correctly and (2) were in disagreement regarding the predicted label. In cases where all models were in agreement on the correct prediction, the validity of the classification would be supported by their focus on relevant tissue regions for diagnosis. For example, in the prediction of MSI status, models predominantly highlighted tumour regions, as expected. However, models such as UNI, Hibou, Virchow and Kaiko occasionally highlighted pen marks, which is an undesired behaviour that suggests that predictions are being made through some form of pattern association rather than understanding the underlying biology (Fig. 4a and Extended Data Fig. 7b). To assess the impact of pen marks, we quantified their occurrence in 50 randomly sampled slides per test cohort and found them present in 90% of slides from DACHS and 22% from Bern, but absent elsewhere. Despite their presence, pen marks did not skew classification, as they were equally distributed across different classes. Models such as CONCH and Virchow focused on multiple small tissue areas, whereas Prov-GigaPath appears less selective in its attention (Fig. 4a). In NSCLC subtyping, models generally performed well, focusing mainly on tumour regions and ignoring healthy lung parenchyma (Extended Data Fig. 8b). In *ESR1* overexpression prediction, Prov-GigaPath and Kaiko highlighted the majority of the WSI area, whereas CONCH and Virchow focused on a few small tissue areas (Extended Data Fig. 8c). By contrast, when analysing slides where models made inconsistent predictions, we found instances of model disagreement that led to errors. For instance, in the task of DACHS CRC sidedness, Virchow erroneously focused on pen marks (Extended Data Fig. 7b). However, no consistent pattern of errors emerged across the models to fully explain these discrepancies.

Together, these data indicate that foundation models vary in their focus on tissue regions and the morphological features that they prioritize, which impacts their predictive performance. The differences in attention across models suggest that combining models with complementary strengths could enhance overall predictive accuracy in ensemble approaches.

### Ensemble of pathology foundation models improve performance

Lastly, we tested the hypothesis that creating an ensemble of pathology foundation models improves prediction performance. We utilized two approaches for ensembling models, taking the average of the various downstream models’ prediction scores trained on different foundation model backbones and concatenating feature vectors from different foundation model backbones to create a single downstream model.

**Extended Data Fig. 9:**
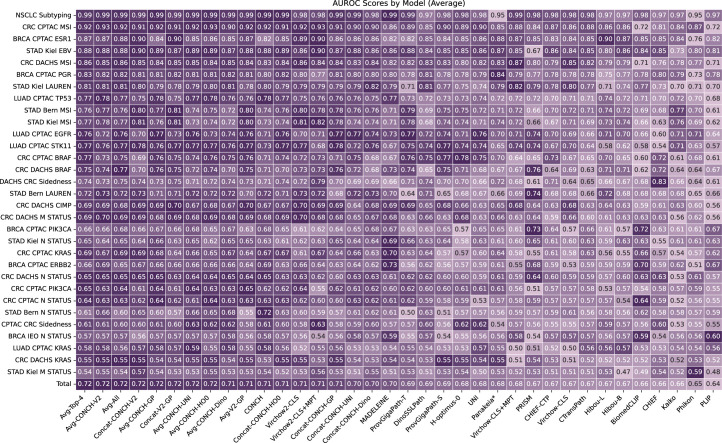
AUROC scores across all foundation models and ensembles. AUROC scores for all foundation models, foundation model variations, and multiple ensemble approaches. Prov-GigaPath-T are the regular tile embeddings, Prov-GigaPath-S are the tile embeddings encoded by the GigaPath slide encoder. Virchow(-2)-CLS contained only class tokens, with Virchow(-2)-CLS + MPT representing the version with class and mean patch tokens combined. Multiple experiments were conducted using concatenated feature vectors combining features from CONCH, Virchow2, Prov-GigaPath, DinoSSLPath, H-optimus-0 and UNI. For the same combinations, average prediction scores were calculated. These scores were used to evaluate the performance of combined predictions. Source data

Experiments show that ensembling by taking the average of the models’ prediction scores yielded a superior AUROC compared with either model used in isolation. The combination of the four top-performing models led to the highest improvement, achieving a mean AUROC 1.2% higher than CONCH (Extended Data Fig. 9), the leading individual model (Fig. 1b). Across all 31 tasks, the ensemble reduced misclassifications compared with CONCH by an average of 6.2% across the five folds (cut-off 0.5) (Supplementary Table 2). Therefore, these data show that ensembling the prediction scores of multiple high-performing models enhances performance on certain tasks beyond the capabilities of the best individual model.

**Extended Data Fig. 10:**
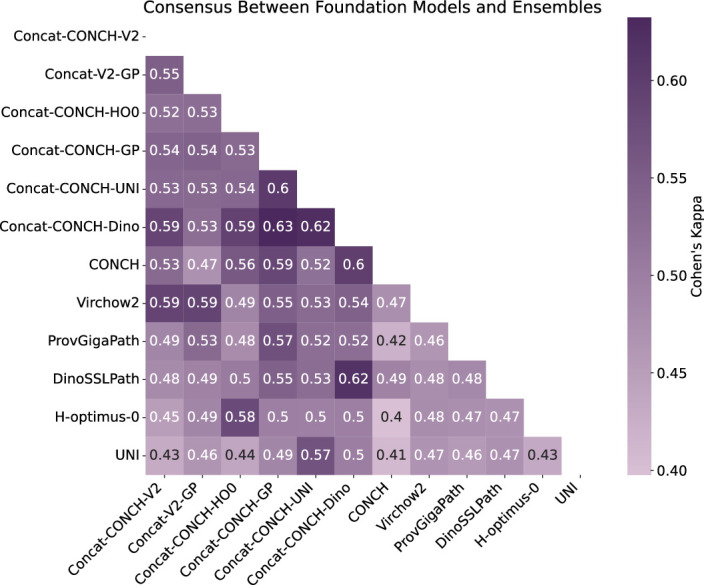
Cohen’s kappa scores across all ensembles and their individual model components. Objective measure of similarity of prediction scores using Cohen’s Kappa and majority vote across the five folds to binarize the predictions. The concatenated versions of CONCH, Virchow2 (V2), Prov-GigaPath (GP), H-optimus-0 (HO0), UNI and DinoSSLPath (Dino) and their single model counterparts are shown. Source data

Combining the best-performing models, CONCH and Virchow2, yielded a 1,792-dimensional vector with the highest AUROC of 71.9. Similarly, combining Virchow2 and Prov-GigaPath, the top-performing vision-only models, resulted in a 2,816-dimensional vector with an AUROC of 71.6. Individually, the models achieved AUROCs of 71.1 for CONCH, 70.9 for Virchow2 and 69.2 for Prov-GigaPath (Fig. 1b and Extended Data Fig. 9). Interestingly, Cohen’s kappa between the individual models did not strongly correlate with ensemble quality, indicating that low agreement does not necessarily translate to beneficial diversity in predictions. Similarly, no clear pattern was observed between the similarity of ensembles with their single model counterparts and factors such as model performance or embedding size (Extended Data Fig. 10). To quantify improvements, we conducted two-sided DeLong’s tests comparing AUROC scores of CONCH with ensembles and other single-model baselines. For each model, we averaged prediction scores across five folds, and across up to ten folds for ensembles. Bagging the five folds of the same foundation model increased AUROC scores, while integrating different models via stacking or concatenation yielded more pronounced improvements (Extended Data Fig. 4a). The CONCH and Virchow2 ensemble showed statistically significant differences in performance with higher AUROCs than CONCH in 9 of 29 tasks (*P* < 0.05), whereas the Virchow2 and Prov-GigaPath ensemble showed significant improvements in 7 tasks (Extended Data Fig. 4b).

These results demonstrate that ensemble approaches for pathology foundation models, as well as their downstream models, lead to enhanced prediction performance. This suggests that merging multiple foundation models through ensemble techniques can be beneficial.

## Discussion

Weakly supervised computational pathology approaches, in which a deep learning system predicts a label directly from a WSI, have been massively successful in cancer research. They have been used to make the diagnosis of tumours, to predict biomarker status and to predict clinical outcomes directly from image data. Over 100 such tools are now approved for clinical use in the United States and the European Union^26,27^. Since 2022, foundation models have become an integral part of weakly supervised computational pathology pipelines and have improved performance and generalizability^4,28^. However, the current internal evaluation strategy for foundation models in computational pathology for clinically relevant tasks is limited. When groups that publish pathology foundation models evaluate them on tasks of their own choosing, there is a high potential for bias. Moreover, concerns about data leakage arise when foundation models are tested on images from the same institutions where they were trained.

In this study, we conducted a comprehensive evaluation of pathology foundation models in weakly supervised computational pathology on truly external datasets with no overlap between training and validation data. Our results show that while many existing foundation models achieve high performance on clinically relevant prediction tasks, CLIP-based approaches are not inherently superior, as evidenced by BiomedCLIP and PLIP’s performance. Instead, high-quality pretraining data and effective data cleaning are crucial for achieving top-tier performance. The best-performing model, CONCH, trained with multimodal data, suggests that incorporating text during training enhances image-only embedding quality. Similarly, Virchow2’s strong performance stems from its unprecedented tissue type diversity (approximately 200 versus 20–30 in other models) and more balanced distribution, avoiding over-representation of specific cancer types. In addition, the variability in the model’s performance can also be attributed to varying degrees of difficulty for each task. For instance, while differentiating between lung carcinoma subtypes is generally straightforward, other tasks such as stomach cancer subtyping can be more demanding. Here even pathologists can show a considerate degree of interobserver disagreement^29^.

In terms of prediction interpretability, our approach highlights that different foundation models focus on different areas in the tissue while still having a high agreement on the predicted label. Our technical analysis revealed that slide encoders showed no advantage over tile encoders in MIL set-ups, except in low-data scenarios, and the transformer-based STAMP architecture generally outperformed ABMIL outside of data-limited settings. Interestingly, while CONCH dominated in tasks when trained on the full dataset, its advantages diminished in low-data and low-prevalence settings. This performance dichotomy suggests that multimodal training of a foundation model, despite its presumed benefits, does not confer special advantages in the data-constrained scenarios often encountered in clinical settings within the scope of our experiments. We demonstrate that ensembling foundation models is beneficial, particularly when combining top-performing models, although prediction diversity (measured by Cohen’s kappa) does not directly correlate with ensemble performance. Even modest ensemble improvements may have clinical relevance by combining several learned perspectives of tissue morphology, as exemplified by the higher biomarker classification performance. Future work should incorporate more sophisticated methods than feature vector concatenation, especially for larger models where combining large vectors might lead to overfitting.

A key insight of our study is that performance of foundation models does not scale well with increasing numbers of images in the training set used for SSL. This means that bigger is not always better. Rather, the diversity of the training set suggests to be a key factor, favouring various sources of data, races and types of cancer. Our results will inform the future development of new foundation models. Specifically, using multimodal data to train models, even if the intention is just to apply them on unimodal data (that is, on images alone), should be encouraged. For healthcare institutions, this means that data that is available at scale, even without clinical association with clinical endpoints, is a valuable resource to train such models. Moreover, our findings suggest that the selected computational pathology tasks may be solvable primarily through local morphological patterns rather than requiring global spatial context. The performance achieved by randomly sampling 512 tiles per patient at each epoch suggests that for many tasks, the discriminative features exist at the local level. This observation is consistent with our comparison showing that tile-level encoders outperformed slide-level encoders despite the latter’s theoretical advantage in capturing global spatial relationships. Future research should explore in further depth whether the selected tasks and performance metrics adequately represent the spectrum of diagnostic challenges, particularly those requiring integration of long-range spatial dependencies across the entire slide.

Our study has limitations in that our evaluation tasks only contain certain tumour types. We focused on four cancer types, prioritizing truly external validation datasets over broader cancer type coverage. This differentiates our work from studies that train and test on the same cohort or WSIs from the same hospital used for pretraining. Moreover, we were limited to pathology foundation models licences, which are accessible in a research setting. For example, this excludes RudolphV and PLUTO from our analysis. While our datasets contained artefacts such as pen marks (present in 90% of DACHS and 22% of Bern samples), these had minimal impact on predictions owing to their even distribution across classes. Although we incorporated a broad range of foundation models applicable to histology data, exploring the potential of fine-tuning general-purpose models such as GPT-4o was outside our current scope. Our evaluation strategy is focused on a diverse set of biomarkers in cancer histopathology. Future work will expand upon the range of tumour types, biomarkers and patient cohorts to further evaluate the robustness of foundation models in pathology.

## Methods

### Ethics statement

This study was carried out in accordance with the Declaration of Helsinki. The Clinical Proteomic Tumor Analysis Consortium (CPTAC) and TCGA did not require formal ethics approval for a retrospective study of anonymized samples. The analysis of the testing cohort DACHS (an epidemiological study that is led by the German Cancer Research Center, DKFZ) was approved by the ethics committee of the Medical Faculty, University of Heidelberg, under 310/2001^30–32^.

### Datasets

The study used datasets from TCGA, CPTAC and proprietary cohorts. Specifically, cohorts from LUAD, lung squamous cell carcinoma (LUSC), CRC, STAD and BRCA were included. TCGA datasets were used for training of the models, and CPTAC, DACHS, Kiel, Bern and IEO were used for evaluation. This ensured that all testing was done on data that had neither been seen during training of the foundation models nor the aggregator models. For our analyses, we only use the CPTAC-2 and CPTAC-3 prospective collections (from 2018/20), which exclusively contain patients with CPTAC-IDs and have no overlap with TCGA patients.

For external validation, CPTAC datasets for LUAD, LUSC, colorectal adenocarcinoma and BRCA were used. No foundation models analysed in this study were trained on CPTAC, ensuring its suitability as an independent test cohort. In addition, for CRC, the DACHS cohort was utilized alongside CPTAC as another external test set. In STAD, proprietary datasets from Kiel and Bern served as external validation cohorts. For BRCA, the IEO dataset was used alongside CPTAC for external validation (Fig. 1a and Supplementary Fig. 2).

### Experimental design

Digital pathology involves several task categories, including morphological, biomarker and prognostic tasks, and foundation models should be capable of performing well across all of them. In this study, we assembled and benchmarked 19 foundation models—the 12 pure vision models CTransPath^28^, DinoSSLPath^33^, Phikon^16^, UNI^21^, Virchow^23^, Kaiko (ViT-L/14)^34^, Prov-GigaPath^22^, Hibou-B, Hibou-L^35^, H-optimus-0^36^, Virchow2^37^ and Panakeia, the 3 vision-language models PLIP^38^, BiomedCLIP^39^ and CONCH^40^, and the 4 slide encoders GigaPath, MADELEINE^41^, PRISM^42^ and CHIEF^43^—across a comprehensive set of tasks from all three categories. Each category was assessed across all cancer types, apart from morphological features in BRCA and prognostic features in NSCLC owing to data unavailability. Biomarkers were selected based on clinical relevance, diversity and availability. Tasks were prioritized when they were associated with actionable therapeutic targets, as annotated by OncoKB^44^. To enable both training and independent testing, each task required ground truth data to be available in TCGA (for training) and at least one test cohort. For each cohort, only tasks with at least ten cases in each category were included (Supplementary Table 3). For visualization purposes, only 15 models (vision-only and vision-language models) are shown in most figures. The slide encoders were included selectively, such as in Fig. 2g for comparison with their tile embedding counterparts and in Fig. 3d,e and Extended Data Fig. 5 to highlight their potential benefits in scarce data settings. Extended Data Figs. 1 and 9 include all models to comprehensively show all experiments.

First, we investigated morphological classification tasks related to cancer subgroups with distinct phenotypic characteristics. The aim was to assess foundation models by evaluating their ability to discern established phenotypic distinctions. In CRC, the morphological task involved predicting whether the slide originated from the left or right side of the colon, excluding colon transversum samples owing to ambiguous classification. In STAD, the Lauren classification^45^ was chosen as the morphological task, classifying slides as ‘intestinal’, ‘diffuse’ or ‘mixed’, given the unavailability of ground truth for newer classification systems^46,47^. In lung cancer, the models were tasked with classifying samples into either adenocarcinoma or squamous cell carcinoma^1^.

Biomarker prediction tasks focused mainly on clinically relevant targets with some type of morphological correlation as demonstrated by previous computational pathology models. For CRC, these included *BRAF*, *KRAS*, MSI status, *PIK3CA* and CIMP status^11^. For STAD, EBV presence and MSI status were selected^48^. For LUAD, the targets were *EGFR*, *STK11*, *KRAS* and *TP53*^1^. For BRCA, the targets were the expression of HER2, ER and PR receptors and *PIK3CA* mutations^49,50^. MSI status and CIMP status were binarized into MSI-high versus not MSI-high and CIMP-high versus not CIMP-high, respectively. *HER2*, *ESR1* and *PGR* expression were binarized using the *z*-score of mRNA expression profiles, similar to a study by Wegscheider et al.^51^. This approach was preferred over immunohistochemistry labels owing to its objectivity and reduced variance error.

Prognostic tasks, which aim to predict clinical outcomes directly from WSIs, were selected based on their prognostic relevance. The tasks included N-status for CRC, STAD and BRCA, where all stages except N0 were classified as N+ (excluding Nx cases). M-status was analysed in CRC and STAD, performing binary classification of M0 versus M+.

By focusing on tasks with clear therapeutic actionability or prognostic relevance, we aimed to evaluate the practical utility of these models in a clinical setting. This comprehensive benchmarking study included 31 tasks across 8 external test cohorts, encompassing a wide range of clinically relevant classification tasks (Supplementary Table 4).

### Image processing and deep learning techniques

The benchmarking was conducted using the STAMP pipeline version 1.1.1 (ref. ^19^) (Supplementary Table 5). Each classification task followed a two-step procedure (Fig. 1b). In the first step, feature vectors were extracted from WSIs utilizing the foundational models evaluated in this study. In the second step, these vectors were used to train a slide-level aggregator on the downstream tasks described above.

WSIs were segmented into N tiles, with an edge length of 224 pixels corresponding to 256 µm, resulting in an effective resolution of ~1.14 µm per pixel. All included foundation models in our benchmark, except for Prov-GigaPath^22^, tessellate the slide into tiles of 224 × 224 pixels. However, the Prov-GigaPath implementation transforms tiles using centre cropping from 256 × 256 into 224 × 224 before inputting it into the tile encoder. The slide encoder then processes these feature embeddings generated by the tile encoder, implicitly maintaining the 224 × 224 tile dimensionality throughout the pipeline. Therefore, our choice of tile dimensionality for slide tessellation is consistent with the foundation models selected for our analyses. Background tiles were excluded using Canny edge detection^52^. Stain normalization was not applied during preprocessing. Feature extraction was performed on each tile individually using the different foundational models. The embedding dimensions *M* varied across models, ranging from *M* = 384 for DinoSSLPath and Panakeia to *M* = 1,536 for Prov-GigaPath and H-optimus-0. Subsequently, each slide was transformed into a two-dimensional matrix with dimension *N* × *M*. The extracted feature vectors were input into a transformer-based aggregator model^4^. It utilizes multi-head attention, Gaussian error linear unit activation functions^53^, layer normalization and a multilayer perceptron (MLP) head to produce an output corresponding to the *k* possible classes for each task. A 5-fold cross-validation approach was implemented, resulting in the creation of 2,945 models (19 foundation models, 31 tasks and 5 folds) trained exclusively on TCGA datasets. We implemented stratified *k*-fold cross-validation to ensure that each fold maintains representative proportions of all classes, preventing scenarios where rare categories have zero instances in training runs. This approach follows standard practices in computational pathology and provides robust performance estimates and better generalization assessment^10^. All experiments were run on individual 40 GB NVIDIA RTX A6000 and L40 GPU (graphics processing unit) nodes. In addition to the transformer-based aggregator described, we evaluated ABMIL as an alternative aggregation method^24^. ABMIL introduces inductive bias by using attention mechanisms to assign weights to each tile in a slide, enabling the model to focus on the most informative regions.

To integrate slide encoders into the MIL pipeline, we extracted the encoded tile-level embeddings for Prov-GigaPath, MADELEINE, CHIEF and the 512 latents for PRISM. These encoded tile embeddings were subsequently treated as regular tile embeddings in all analyses. Unless explicitly stated otherwise, results presented throughout the study refer to the regular tile embeddings. Prov-GigaPath provides both a slide-level and a tile-level encoder, and we evaluated both approaches^22^. In the case of Virchow and Virchow2, Vorontsov et al. proposed concatenating the class token with the average pool of patch tokens for each tile embedding. To maintain consistency with other models that only use class tokens, two configurations were tested: one including and one excluding the averaged patch tokens. As the differences are very small, the version only using class tokens is shown in the main results for consistency with other models. For CONCH, we used the output of the attentional pooler that corresponds to image-text alignment, with an embedding dimension of 512. Although the Panakeia models are specifically designed for BRCA and CRC, respectively, we also evaluate the CRC model on STAD and the BRCA model on NSCLC. This is because their performance remains competitive in these contexts, and including these results provides the basis for comparison in subsequent analyses. For experiments involving combined feature vectors, vectors were concatenated, maintaining a single vector per tile. For instance, combining CONCH and Virchow2 resulted in a combined embedding dimension *M* of *M* = 1,792 (*M* = 512 for CONCH + *M* = 1,280 for Virchow2).

### Explainability

To better interpret the output of the models, we generated whole-slide prediction heatmaps for selected tasks. These heatmaps illustrate the models’ focus on specific tissue areas, by weighting the scores assigned to individual tiles using gradient-weighted class activation mapping (Grad-CAM)^54^. It is important to note that a high number of positively contributing tiles do not automatically result in a high final score owing to the nonlinear aggregation process in neural networks^55^. The benchmarking effort involved 2,945 models and 9,528 slides, leading to a vast number of model-slide combinations. Thus, it was necessary to select a few informative examples methodically. Slides were selected by including cases where models showed strong disagreements and cases where all models performed well. The heatmaps were visually analysed and compared with the underlying WSI. To further analyse the similarity between different models, Cohen’s kappa^25^ was measured between each pair of foundation models.

### Statistical analysis

The performance of the models was evaluated using the AUROC using fivefold cross-validation and deployment on external cohorts. Mean AUROC scores from the five cross-validation models deployed on external data were used for statistical and graphical evaluations. Predictions were made per patient, and all feature matrices belonging to one patient were concatenated for use in the model. In addition to AUROC, for completeness in the supplementary material, we also calculated the AUPRC, balanced accuracy and F1 scores. The two-sided DeLong’s test was used to test for statistically significant differences in AUROC scores. As the DeLong’s test is only applicable when a single prediction score is available for each model and sample, the average prediction score across all five folds was used. Owing to its multi-class nature, we excluded Lauren classification tasks from this analysis. This differs from the main metrics, where the AUROC/AUPRC/F1/balanced accuracy scores represent the mean across the five folds.

### Reporting summary

Further information on research design is available in the Nature Portfolio Reporting Summary linked to this article.

## Supplementary information

Supplementary Information Supplementary Tables 1–8, methods and Figs. 1–4.

Reporting Summary

Peer Review File

## Source data

Source Data Fig. 1 Statistical source data. An Excel file containing the numerical data used to generate all graphs and heatmaps in the corresponding figure. The data are organized in separate worksheets that correspond to each figure panel.

Source Data Fig. 2 Statistical source data. An Excel file containing the numerical data used to generate all graphs and heatmaps in the corresponding figure. The data are organized in separate worksheets that correspond to each figure panel.

Source Data Fig. 3 Statistical source data. An Excel file containing the numerical data used to generate all graphs and heatmaps in the corresponding figure. The data are organized in separate worksheets that correspond to each figure panel.

Source Data Fig. 4 Statistical source data. An Excel file containing the numerical data used to generate all graphs and heatmaps in the corresponding figure. The data are organized in separate worksheets that correspond to each figure panel.

Source Data Extended Data Fig. 1 Statistical source data. An Excel file containing the numerical data used to generate all graphs and heatmaps in the corresponding figure. The data are organized in separate worksheets that correspond to each figure panel.

Source Data Extended Data Fig. 2 Statistical source data. An Excel file containing the numerical data used to generate all graphs and heatmaps in the corresponding figure. The data are organized in separate worksheets that correspond to each figure panel.

Source Data Extended Data Fig. 3 Statistical source data. An Excel file containing the numerical data used to generate all graphs and heatmaps in the corresponding figure. The data are organized in separate worksheets that correspond to each figure panel.

Source Data Extended Data Fig. 4 Statistical source data. An Excel file containing the numerical data used to generate all graphs and heatmaps in the corresponding figure. The data are organized in separate worksheets that correspond to each figure panel.

Source Data Extended Data Fig. 5 Statistical source data. An Excel file containing the numerical data used to generate all graphs and heatmaps in the corresponding figure. The data are organized in separate worksheets that correspond to each figure panel.

Source Data Extended Data Fig. 6 Statistical source data. An Excel file containing the numerical data used to generate all graphs and heatmaps in the corresponding figure. The data are organized in separate worksheets that correspond to each figure panel.

Source Data Extended Data Fig. 9 Statistical source data. An Excel file containing the numerical data used to generate all graphs and heatmaps in the corresponding figure. The data are organized in separate worksheets that correspond to each figure panel.

Source Data Extended Data Fig. 10 Statistical source data. An Excel file containing the numerical data used to generate all graphs and heatmaps in the corresponding figure. The data are organized in separate worksheets that correspond to each figure panel.

## Data Availability

The slides for TCGA are available at https://portal.gdc.cancer.gov/. The slides for CPTAC are available at https://proteomics.cancer.gov/data-portal. The molecular data for TCGA and CPTAC are available at https://www.cbioportal.org/. The slides and biomarker data for DACHS were generated for previous studies^56–58^ with restricted access. Biomarker data for DACHS are available by requesting Authorized Access to the phs001078 study (https://www.ncbi.nlm.nih.gov/projects/gap/cgi-bin/study.cgi?study_id=phs001113.v1.p1). Applications for access to DACHS biomarker data are reserved for senior investigators and NIH investigators as defined in https://dbgap.ncbi.nlm.nih.gov/aa/wga.cgi, and upon successful application grants access to the data for 1 year with the option to renew access. The slides for DACHS can only be requested directly through the DACHS principal investigators. The contact details are listed at http://dachs.dkfz.org/dachs/kontakt.html. The Kiel cohort is available from the Department of Pathology, Christian Albrechts University of Kiel, Kiel, Germany, upon reasonable request (https://www.medizin.uni-kiel.de/en/institutes-departments/institutes-of-clinical-theory/department-of-pathology). The Bern cohort is proprietary and cannot be shared at the individual patient level. It is archived at the Institute of Pathology, University of Bern, and can be requested in reference to ref. ^59^. The IEO cohort is held by the European Institute of Oncology, Milan. Data requests will be evaluated on a case-by-case basis in accordance with institutional policies and privacy regulations and can be directed via https://www.ieo.it/en/contact_us/. Source data are provided with this paper.

## Code availability

The benchmarking experiments were built upon the open-source STAMP software. All public models tested in this study are available via Zenodo at 10.5281/zenodo.15749283 (ref. ^60^).

## References

[1] Coudray, N. et al. Classification and mutation prediction from non-small cell lung cancer histopathology images using deep learning. *Nat. Med.* **24**, 1559–1567 (2018). 30224757 10.1038/s41591-018-0177-5 PMC9847512

[2] Kather, J. N. et al. Deep learning can predict microsatellite instability directly from histology in gastrointestinal cancer. *Nat. Med.* **25**, 1054–1056 (2019). 31160815 10.1038/s41591-019-0462-y PMC7423299

[3] Lu, M. Y. et al. Data-efficient and weakly supervised computational pathology on whole-slide images. *Nat. Biomed. Eng.* **5**, 555–570 (2021). 33649564 10.1038/s41551-020-00682-w PMC8711640

[4] Wagner, S. J. et al. Transformer-based biomarker prediction from colorectal cancer histology: a large-scale multicentric study. *Cancer Cell* **41**, 1650–1661.e4 (2023). 37652006 10.1016/j.ccell.2023.08.002 PMC10507381

[5] Loeffler, C. M. L. et al. Prediction of homologous recombination deficiency from routine histology with attention-based multiple instance learning in nine different tumor types. *BMC Biol.* **22**, 225 (2024). 39379982 10.1186/s12915-024-02022-9 PMC11462727

[6] Liu, Q. et al. Identification of lymph node metastasis in pre-operation cervical cancer patients by weakly supervised deep learning from histopathological whole-slide biopsy images. *Cancer Med.* **12**, 17952–17966 (2023). 37559500 10.1002/cam4.6437 PMC10523985

[7] da Silva, L. M. et al. Independent real-world application of a clinical-grade automated prostate cancer detection system. *J. Pathol.* **254**, 147–158 (2021). 33904171 10.1002/path.5662 PMC8252036

[8] Bagg, A. et al. Performance evaluation of a novel artificial intelligence-assisted digital microscopy system for the routine analysis of bone marrow aspirates. *Mod. Pathol.* **37**, 100542 (2024). 38897451 10.1016/j.modpat.2024.100542

[9] Yang, Z. et al. The devil is in the details: a small-lesion sensitive weakly supervised learning framework for prostate cancer detection and grading. *Virchows Arch.* **482**, 525–538 (2023). 36823229 10.1007/s00428-023-03502-z

[10] El Nahhas, O. S. M. et al. Regression-based deep-learning predicts molecular biomarkers from pathology slides. *Nat. Commun.* **15**, 1253 (2024). 38341402 10.1038/s41467-024-45589-1 PMC10858881

[11] Niehues, J. M. et al. Generalizable biomarker prediction from cancer pathology slides with self-supervised deep learning: a retrospective multi-centric study. *Cell Rep. Med.* **4**, 100980 (2023). 36958327 10.1016/j.xcrm.2023.100980 PMC10140458

[12] Moor, M. et al. Foundation models for generalist medical artificial intelligence. *Nature* **616**, 259–265 (2023). 37045921 10.1038/s41586-023-05881-4

[13] Waqas, A. et al. Revolutionizing digital pathology with the power of generative artificial intelligence and foundation models. *Lab. Invest.* **103**, 100255 (2023). 37757969 10.1016/j.labinv.2023.100255

[14] He, K., Fan, H., Wu, Y., Xie, S. & Girshick, R. B. Momentum contrast for unsupervised visual representation learning. In *Proc. IEEE Computer Society Conference on Computer Vision and Pattern Recognition* 9726–9735 (IEEE, 2020).

[15] Chen, T., Kornblith, S., Norouzi, M. & Hinton, G. A simple framework for contrastive learning of visual representations. In *Proc. 37th International Conference on Machine Learning* (eds Daumé, H. III & Singh, A.) Vol. 119, 1597–1607 (PMLR, 2020).

[16] Filiot, A. et al. Scaling self-supervised learning for histopathology with masked image modeling. Preprint at *medRxiv*10.1101/2023.07.21.23292757 (2023).

[17] Wu, W., Gao, C., DiPalma, J., Vosoughi, S. & Hassanpour, S. Improving representation learning for histopathologic images with cluster constraints. In *Proc. IEEE International Conference on Computer Vision* 21347–21357 (IEEE, 2023). 10.1109/iccv51070.2023.01957 PMC11062482 38694561

[18] Dosovitskiy, A. et al. An image is worth 16×16 words: transformers for image recognition at scale. In *Proc. International Conference on Learning Representations* (ICLR, 2021).

[19] El Nahhas, O. S. M. et al. From whole-slide image to biomarker prediction: end-to-end weakly supervised deep learning in computational pathology. *Nat. Protoc.* **20**, 293–316 (2024). 39285224 10.1038/s41596-024-01047-2

[20] Schömig-Markiefka, B. et al. Quality control stress test for deep learning-based diagnostic model in digital pathology. *Mod. Pathol.* **34**, 2098–2108 (2021). 34168282 10.1038/s41379-021-00859-x PMC8592835

[21] Chen, R. J. et al. Towards a general-purpose foundation model for computational pathology. *Nat. Med.* **30**, 850–862 (2024). 38504018 10.1038/s41591-024-02857-3 PMC11403354

[22] Xu, H. et al. A whole-slide foundation model for digital pathology from real-world data. *Nature* **630**, 181–188 (2024). 38778098 10.1038/s41586-024-07441-w PMC11153137

[23] Vorontsov, E., Bozkurt, A., Casson, A. & Shaikovski, G. A foundation model for clinical-grade computational pathology and rare cancers detection. *Nat. Med.* **30**, 2924–2935 (2024). 39039250 10.1038/s41591-024-03141-0 PMC11485232

[24] Ilse, M., Tomczak, J. M. & Welling, M. Attention-based deep multiple instance learning. In *Proc. 35th International Conference on Machine Learning* Vol*.* 80, 2127–2136 (PMLR, 2018)

[25] Cohen, J. A coefficient of agreement for nominal scales. *Educ. Psychol. Meas.* **20**, 37–46 (1960).

[26] Geaney, A. et al. Translation of tissue-based artificial intelligence into clinical practice: from discovery to adoption. *Oncogene* **42**, 3545–3555 (2023). 37875656 10.1038/s41388-023-02857-6 PMC10673711

[27] Benjamens, S., Dhunnoo, P. & Meskó, B. The state of artificial intelligence-based FDA-approved medical devices and algorithms: an online database. *npj Digit. Med.* **3**, 118 (2020). 32984550 10.1038/s41746-020-00324-0 PMC7486909

[28] Wang, X. et al. Transformer-based unsupervised contrastive learning for histopathological image classification. *Med. Image Anal.* **81**, 102559 (2022). 35952419 10.1016/j.media.2022.102559

[29] Palli, D. et al. Reproducibility of histologic classification of gastric cancer. *Br. J. Cancer* **63**, 765–768 (1991). 2039701 10.1038/bjc.1991.171 PMC1972379

[30] Carr, P. R. et al. Estimation of absolute risk of colorectal cancer based on healthy lifestyle, genetic risk, and colonoscopy status in a population-based study. *Gastroenterology* **159**, 129–138.e9 (2020). 32179093 10.1053/j.gastro.2020.03.016 PMC7387145

[31] Hoffmeister, M. et al. Colonoscopy and reduction of colorectal cancer risk by molecular tumor subtypes: a population-based case-control study. *Am. J. Gastroenterol.* **115**, 2007–2016 (2020). 32858564 10.14309/ajg.0000000000000819

[32] Brenner, H., Chang-Claude, J., Seiler, C. M., Stürmer, T. & Hoffmeister, M. Does a negative screening colonoscopy ever need to be repeated? *Gut* **55**, 1145–1150 (2006). 16469791 10.1136/gut.2005.087130 PMC1856263

[33] Kang, M., Song, H., Park, S., Yoo, D. & Pereira, S. Benchmarking self-supervised learning on diverse pathology datasets. In *IEEE/CVF Conference on Computer Vision and Pattern Recognition* 3344–3354 (IEEE, 2023).

[34] Ai, K. et al. Towards large-scale training of pathology foundation models. Preprint at 10.48550/arXiv.2404.15217 (2024).

[35] Nechaev, D., Pchelnikov, A. & Ivanova, E. Hibou: a family of foundational vision transformers for pathology. Preprint at 10.48550/arXiv.2406.05074 (2024).

[36] Saillard, C. et al. H*-*optimus-0 https://github.com/bioptimus/releases/tree/main/models/h-optimus/v0 (2024).

[37] Zimmermann, E. et al. Virchow2: scaling self-supervised mixed magnification models in pathology. Preprint at 10.48550/arXiv.2408.00738 (2024).

[38] Huang, Z., Bianchi, F., Yuksekgonul, M., Montine, T. J. & Zou, J. A visual-language foundation model for pathology image analysis using medical Twitter. *Nat. Med.* **29**, 2307–2316 (2023). 37592105 10.1038/s41591-023-02504-3

[39] Zhang, S. et al. BiomedCLIP: a multimodal biomedical foundation model pretrained from fifteen million scientific image–text pairs. *NEJM AI* **2**, AIoa2400640 (2025).

[40] Lu, M. Y. et al. A visual-language foundation model for computational pathology. *Nat. Med.* **30**, 863–874 (2024). 38504017 10.1038/s41591-024-02856-4 PMC11384335

[41] Jaume, G. et al. Multistain pretraining for slide representation learning in pathology. In *Computer Vision – ECCV 2024. Lecture Notes in Computer Science* (eds Leonardis, A. et al.) Vol. 15091, 19–37 (Springer, 2025).

[42] Shaikovski, G. et al. PRISM: a multi-modal generative foundation model for slide-level histopathology. Preprint at 10.48550/arXiv.2405.10254 (2024).

[43] Wang, X. et al. A pathology foundation model for cancer diagnosis and prognosis prediction. *Nature* **634**, 970–978 (2024). 39232164 10.1038/s41586-024-07894-z PMC12186853

[44] Chakravarty, D. et al. OncoKB: a precision oncology knowledge base. *JCO Precis. Oncol.* **2017**, PO.17.00011 (2017). 10.1200/PO.17.00011 PMC5586540 28890946

[45] Lauren, P. The two histological main types of gastric carcinoma: diffuse and so-called intestinal-type carcinoma. An attempt at a histo-clinical classification. *Acta Pathol. Microbiol. Scand.* **64**, 31–49 (1965). 14320675 10.1111/apm.1965.64.1.31

[46] Veldhuizen, G. P. et al. Deep learning-based subtyping of gastric cancer histology predicts clinical outcome: a multi-institutional retrospective study. *Gastric Cancer* **26**, 708–720 (2023). 37269416 10.1007/s10120-023-01398-x PMC10361890

[47] Nagtegaal, I. D. et al. The 2019 WHO classification of tumours of the digestive system. *Histopathology* **76**, 182–188 (2020). 31433515 10.1111/his.13975 PMC7003895

[48] Muti, H. S. et al. Development and validation of deep learning classifiers to detect Epstein-Barr virus and microsatellite instability status in gastric cancer: a retrospective multicentre cohort study. *Lancet Digit. Health* **3**, e654–e664 (2021). 34417147 10.1016/S2589-7500(21)00133-3 PMC8460994

[49] Kather, J. N. et al. Pan-cancer image-based detection of clinically actionable genetic alterations. *Nat. Cancer* **1**, 789–799 (2020). 33763651 10.1038/s43018-020-0087-6 PMC7610412

[50] Mandair, D., Reis-Filho, J. S. & Ashworth, A. Biological insights and novel biomarker discovery through deep learning approaches in breast cancer histopathology. *npj Breast Cancer* **9**, 21 (2023). 37024522 10.1038/s41523-023-00518-1 PMC10079681

[51] Wegscheider, A.-S. et al. Comprehensive and accurate molecular profiling of breast cancer through mRNA expression of *ESR1*, *PGR*, *ERBB2*, *MKI67*, and a novel proliferation signature. *Diagnostics* **14**, 241 (2024). 10.3390/diagnostics14030241 PMC10855423 38337757

[52] Canny, J. A computational approach to edge detection. *IEEE Trans. Pattern Anal. Mach. Intell.* **8**, 679–698 (1986). 21869365

[53] Hendrycks, D. & Gimpel, K. Gaussian error linear units (GELUs). Preprint at 10.48550/arXiv.1606.08415 (2016).

[54] Selvaraju, R. R. et al. Grad-CAM: visual explanations from deep networks via gradient-based localization. *Int. J. Comput. Vis.* **128**, 336–359 (2020).

[55] Chen, R. J. & Krishnan, R. G. Self-supervised vision transformers learn visual concepts in histopathology. Preprint at 10.48550/arXiv.2203.00585 (2022).

[56] Lilla, C. et al. Effect of NAT1 and NAT2 genetic polymorphisms on colorectal cancer risk associated with exposure to tobacco smoke and meat consumption. *Cancer Epidemiol. Biomarkers Prev.* **15**, 99–107 (2006). 16434594 10.1158/1055-9965.EPI-05-0618

[57] Brenner, H., Chang-Claude, J., Seiler, C. M. & Hoffmeister, M. Long-term risk of colorectal cancer after negative colonoscopy. *J. Clin. Oncol.* **29**, 3761–3767 (2011). 21876077 10.1200/JCO.2011.35.9307

[58] Hoffmeister, M. et al. Statin use and survival after colorectal cancer: the importance of comprehensive confounder adjustment. *J. Natl Cancer Inst.* **107**, djv045 (2015). 25770147 10.1093/jnci/djv045

[59] Dislich, B. et al. Preservation of Epstein–Barr virus status and mismatch repair protein status along the metastatic course of gastric cancer. *Histopathology* **76**, 740–747 (2020). 31898331 10.1111/his.14059

[60] Neidlinger, P. et al. STAMP-Benchmark. Source code. *Zenodo*10.5281/zenodo.15749283 (2025).

