# Supplementary material for: Benchmarking foundation models as feature extractors for weakly supervised computational pathology
Source: Nat Biomed Eng. 2025 Oct 1;10(6):1113–23. doi: 10.1038/s41551-025-01516-3 (PMC13279263; doi:10.1038/s41551-025-01516-3)
Supplement: Supplementary file 1 — Supplementary Tables 1–8, methods and Figs. 1–4. [file 41551_2025_1516_MOESM1_ESM.pdf]

nature biomedical engineering

Supplementary information https://doi.org/10.1038/s41551-025-01516-3

Benchmarking foundation models as feature extractors for weakly supervised computational pathology

In the format provided by the authors and unedited

Supplementary Tables

Supplementary Table 1: Analysis of Diversity Metrics in the pretraining datasets

Model	Shannon Entropy	Simpson's Diversity Index	Evenness
UNI	4.03	0.93	0.93
Prov-GigaPath	2.21	0.69	0.57
Virchow	3.31	0.86	0.81
Virchow2	3.97	0.92	0.86

Shannon Entropy: A measure of the uncertainty or randomness in the data distribution. Higher values indicate more diversity.

Simpson's Diversity Index: Measures the probability that two individuals randomly selected from a sample will belong to different categories. Higher values indicate more diversity.

Evenness: Indicates how evenly the data are distributed across categories. It is derived from the Shannon Entropy and ranges from 0 (low evenness) to 1 (high evenness).

These analyses indicate that the UNI model has a more diversified dataset compared to Prov-GigaPath and Virchow, which might have implications for the robustness and generalizability of models trained on these datasets.

Supplementary Table 2: Accuracy and error rates of CONCH versus ensemble model across clinical tasks

	CONCH Accuracy (%)	CONCH Errors (n)	Ensemble Accuracy (%)	Ensemble Errors (n)
NSCLC Subtyping	95%	11	95%	11
CPTAC LUAD EGFR	58%	44	72%	30
CPTAC LUAD KRAS	63%	39	62%	40
CPTAC LUAD STK11	53%	49	75%	27
CPTAC LUAD TP53	71%	31	68%	34
BERN STAD LAUREN	55%	135	55%	138
BERN STAD MSI	84%	48	87%	38
BERN STAD N-Status	69%	93	57%	132
KIEL STAD LAUREN	60%	113	58%	118
KIEL STAD EBV	92%	26	94%	19
KIEL STAD MSI	77%	73	85%	48
KIEL STAD N-Status	63%	119	59%	132
KIEL STAD M-Status	68%	104	72%	88
CPTAC BRCA ERBB2	83%	21	83%	21
CPTAC BRCA ESR1	69%	37	80%	24
CPTAC BRCA PGR	64%	44	67%	39
CPTAC BRCA PIK3CA	69%	37	68%	38
IEO BRCA N-Status	55%	203	56%	198
CPTAC CRC Sidedness	55%	49	53%	51
CPTAC CRC MSI	76%	25	67%	35
CPTAC CRC BRAF	47%	56	39%	65
CPTAC CRC KRAS	65%	37	70%	32
CPTAC CRC PIK3CA	47%	56	48%	55
CPTAC CRC N-Status	59%	45	60%	44
DACHS CRC Sidedness	58%	1028	54%	1123
DACHS CRC MSI	89%	228	90%	199
DACHS CRC BRAF	66%	702	85%	314
DACHS CRC KRAS	41%	1233	36%	1321
DACHS CRC CIMP	77%	515	84%	365
DACHS CRC N-Status	60%	941	60%	943
DACHS CRC M-Status	81%	334	80%	355
**Sum**	**67%**	**6478**	**69%**	**6076**

Accuracy and misclassification counts for CONCH and an ensemble across clinical tasks. The Ensemble method combines the average predictions of CONCH, Virchow2, Prov-Gigapath, and DinoSSLPath. The reported numbers are averaged across five cross-validation folds.

Supplementary Table 3: Clinically relevant tasks excluded due to few cases

STAD				
**Marker**	**Value**	**Dataset**	**Cohort**	**Count**
NTRK1	WT	train	TCGA	321
NTRK1	MUT	train	TCGA	5
EBV	negative	test	Bern	299
EBV	positive	test	Bern	8
M_STATUS	M0	test	Bern	306
M_STATUS	M+	test	Bern	1
**LUAD**				
**Marker**	**Value**	**Dataset**	**Cohort**	**Count**
BRAF*	WT	train	TCGA	432
BRAF*	MUT	train	TCGA	29
BRAF	WT	test	CPTAC	103
BRAF	MUT	test	CPTAC	3
MET*	WT	train	TCGA	441
MET*	MUT	train	TCGA	20
MET	WT	test	CPTAC	106
MET	MUT	test	CPTAC	0
**CRC**				
**Marker**	**Value**	**Dataset**	**Cohort**	**Count**
NRAS*	WT	train	TCGA	529
NRAS*	MUT	train	TCGA	29
NRAS	WT	test	CPTAC	100
NRAS	MUT	test	CPTAC	6

*no external validation tcohort with at least 10 samples

Supplementary Table 4: Patient numbers for individual experiments

CRC				
**Marker**	**Value**	**Dataset**	**Cohort**	**Count**
CRC Sidedness	left	train	TCGA	230
CRC Sidedness	right	train	TCGA	168
MSI	nonMSIH	train	TCGA	368
MSI	MSIH	train	TCGA	61
BRAF	WT	train	TCGA	450
BRAF	MUT	train	TCGA	51
KRAS	WT	train	TCGA	296
KRAS	MUT	train	TCGA	205
CIMP	nonCIMPH	train	TCGA	375
CIMP	CIMPH	train	TCGA	54
PIK3CA	WT	train	TCGA	377
PIK3CA	MUT	train	TCGA	124
N_STATUS	N0	train	TCGA	318
N_STATUS	N+	train	TCGA	238
M_STATUS	M0	train	TCGA	417
M_STATUS	M+	train	TCGA	76
CRC Sidedness	left	test	Dachs	1607
CRC Sidedness	right	test	Dachs	819
MSI	nonMSIH	test	Dachs	1836
MSI	MSIH	test	Dachs	210
BRAF	WT	test	Dachs	1930
BRAF	MUT	test	Dachs	151
KRAS	WT	test	Dachs	1397
KRAS	MUT	test	Dachs	677
CIMP	nonCIMPH	test	Dachs	1878
CIMP	CIMPH	test	Dachs	362
N_STATUS	N0	test	Dachs	1295
N_STATUS	N+	test	Dachs	1085
M_STATUS	M0	test	Dachs	1459
M_STATUS	M+	test	Dachs	337
CRC Sidedness	right	test	CPTAC	57
CRC Sidedness	left	test	CPTAC	51
MSI	nonMSIH	test	CPTAC	81
MSI	MSIH	test	CPTAC	24
BRAF	WT	test	CPTAC	91
BRAF	MUT	test	CPTAC	15
KRAS	WT	test	CPTAC	71
KRAS	MUT	test	CPTAC	35
PIK3CA	WT	test	CPTAC	87
PIK3CA	MUT	test	CPTAC	19
N_STATUS	N0	test	CPTAC	56

N_STATUS	N+	test	CPTAC	54
**STAD**				
**Marker**	**Value**	**Dataset**	**Cohort**	**Count**
LAUREN	intestinal	train	TCGA	148
LAUREN	diffuse	train	TCGA	61
LAUREN	mixed	train	TCGA	10
EBV	negative	train	TCGA	300
EBV	positive	train	TCGA	26
MSI	nonMSIH	train	TCGA	270
MSI	MSIH	train	TCGA	56
N_STATUS	N+	train	TCGA	225
N_STATUS	N0	train	TCGA	97
M_STATUS	M0	train	TCGA	289
M_STATUS	M+	train	TCGA	21
LAUREN	intestinal	test	Bern	172
LAUREN	diffuse	test	Bern	78
LAUREN	mixed	test	Bern	54
MSI	nonMSIH	test	Bern	261
MSI	MSIH	test	Bern	43
N_STATUS	N+	test	Bern	205
N_STATUS	N0	test	Bern	99
LAUREN	intestinal	test	Kiel	187
LAUREN	diffuse	test	Kiel	75
LAUREN	mixed	test	Kiel	20
EBV	negative	test	Kiel	302
EBV	positive	test	Kiel	18
MSI	nonMSIH	test	Kiel	293
MSI	MSIH	test	Kiel	27
N_STATUS	N+	test	Kiel	222
N_STATUS	N0	test	Kiel	98
M_STATUS	M0	test	Kiel	259
M_STATUS	M+	test	Kiel	61
**LUAD**				
**Marker**	**Value**	**Dataset**	**Cohort**	**Count**
EGFR	WT	train	TCGA	411
EGFR	MUT	train	TCGA	50
KRAS	WT	train	TCGA	317
KRAS	MUT	train	TCGA	144
STK11	WT	train	TCGA	394
STK11	MUT	train	TCGA	67
TP53	MUT	train	TCGA	239
TP53	WT	train	TCGA	222
EGFR	WT	test	CPTAC	72
EGFR	MUT	test	CPTAC	34

KRAS	WT	test	CPTAC	74
KRAS	MUT	test	CPTAC	32
STK11	WT	test	CPTAC	88
STK11	MUT	test	CPTAC	18
TP53	MUT	test	CPTAC	55
TP53	WT	test	CPTAC	51
**NSCLC**				
**Marker**	**Value**	**Dataset**	**Cohort**	**Count**
NSCLC Subtyping	AC	train	TCGA	461
NSCLC Subtyping	SCC	train	TCGA	462
NSCLC Subtyping	AC	test	CPTAC	106
NSCLC Subtyping	SCC	test	CPTAC	108
**BRCA**				
**Marker**	**Value**	**Dataset**	**Cohort**	**Count**
ERBB2	negative	train	TCGA	916
ERBB2	positive	train	TCGA	125
ESR1	positive	train	TCGA	770
ESR1	negative	train	TCGA	271
PGR	positive	train	TCGA	704
PGR	negative	train	TCGA	337
PIK3CA	WT	train	TCGA	687
PIK3CA	MUT	train	TCGA	336
N_STATUS	N+	train	TCGA	554
N_STATUS	N0	train	TCGA	468
ERBB2	negative	test	CPTAC	106
ERBB2	positive	test	CPTAC	14
ESR1	positive	test	CPTAC	79
ESR1	negative	test	CPTAC	41
PGR	positive	test	CPTAC	70
PGR	negative	test	CPTAC	50
PIK3CA	WT	test	CPTAC	82
PIK3CA	MUT	test	CPTAC	38
N_STATUS	N+	test	IEO	244
N_STATUS	N0	test	IEO	207

Supplementary Table 5: STAMP hyperparameters

Hyperparameter	Value
Layers	2
Attention heads	8
Head activation	GELU
Embedding dimension (input)	384 to 1536
Embedding dimension (reduced)	512
MLP dimension	512
Drop path rate (Dropout)	0
Weight decay	0.01
Optimizer	AdamW
Learning rate	0.0001
Learning rate schedule	FastAI fit_one_cycle
Float precision	Float32
Batch size (training)	64
Bag size	512
Batch size (validation/testing)	1
Training epochs	32
Early stopping patience	16 epochs without improvement in AUROC
Random seed	Hard-coded

Supplementary Table 6: Models' architecture overview

Name	Released	SSL	Architecture	Pretraining Tile size (px)	Patch token size (px)	Magnification	Embed dim	Dataset	Special attributes
CTransPath	Dec 2021	SRCL	CNN + Swin-Transformer	1024	4	20x	768	TCGA, PAIP	Mean of all tokens as embbedding
DinoSSLPath	Dec 2022	DINO v1	ViT-Small	512	16	20x, 40x	384	TCGA, TULIP	Another DinoSSLPath model with patch token size 8
BiomedCLIP	Mar 2023	CLIP	ViT-Base	224	16	diverse	512	OpenPath	Vision language model
Phikon	Jul 2023	iBOT	ViT-Base	224	16	20x	768	TCGA	
CONCH	Jul 2023	iBOT + CoCa	ViT-Base	256	16	diverse	512	MGH, PMC-Path, EDU	Vision language model
PLIP	Aug 2023	CLIP	ViT-Base	224	32	diverse	512	PMC-15M	Vision language model
UNI	Aug 2023	DINO v2	ViT-Large	256 & 512	16	20x	1024	BWH, MGH, GTEx	
Virchow	Sep 2023	DINO v2	ViT-Huge	224	14	20x	1280/2560	MSKCC	Mean patch tokens added to the tile embeddings
Kaiko	Mar 2024	DINO v2	ViT-Large	256	14	5x,10x,20x,40x	1024	TCGA	
Prov-GigaPath	May 2024	DINO v2	ViT-Giant	256	14	20x	1536	Providence	LongNet slide encoder to further preprocess tile embeddings
Hibou-B	Jun 2024	DINO v2	ViT-Base	?	16	?	768	proprietary	
Hibou-L	Jun 2024	DINO v2	ViT-Large	?	16	?	1024	proprietary	
H-optimus-0	Jul 2024	DINO v2/iBOT	ViT-Giant	224	14	20x	1536	proprietary	
Virchow 2	Aug 2024	DINO v2 (+ ECT and KDE)	ViT-Huge	224	14	5x,10x,20x,40x	1280/2560	MSKCC and diverse international institutions	Also Virchow2G model with a ViT-Giant architecture

Panakeia	-	?	ViT-Small	224	16	?	384	proprietary	Specific cancer models only for BRCA and CRC

Supplementary Table 7: Models' pretraining dataset composition

Name	WSIs (K)	Tiles (M)	Patients (K)	Cancer subtypes	Anatomic sites/Organs	malignant WSIs
CTransPath	32	16	~13	32	25	100%
DinoSSLPath	37	33	?	?	?	?
BiomedCLIP	15,000*	-	?	?	?	?
Phikon	6	43	5.6	16	13	100%
PLIP	208*	-	?	?	?	?
CONCH	1,200*	-	?	350	?	?
UNI	100	100	?	?	20	?
Virchow	1,488	2,000	120	?	17	38%
Kaiko	29	256	11	32	25	100%
Prov-GigaPath	171	1,385	30	?	31	?
Hibou-B	1,139	512	306	?	?	?
Hibou-L	1,139	1,200	306	?	?	?
H-optimus-0	500	"several hundreds of millions"	~333	?	?	?
Virchow2	3,135	1,700	225	?	~175	40%
Panakeia-BRCA	5	13	?	?	1	?
Panakeia-CRC	1	4.5	?	?	1	?

*image-caption pairs

Supplementary Table 8: Proportion of analyzed tissue types in the pretraining data

<table>
<tr><th>Foundation Model</th><th>Number represents</th><th>Lung</th><th>Breast</th><th>Stomach</th><th>Colon</th></tr>
<tr><td>Prov-GigaPath</td><td>Tissue slides</td><td>45%</td><td>2.7%</td><td>0.7%</td><td>30%</td></tr>
<tr><td>CONCH</td><td>Image-text pairs</td><td>103k (9.5%)</td><td>65k (5.6%)</td><td colspan="2">121k (10.4%)</td></tr>
<tr><td>UNI</td><td>Tissue slides</td><td>9846 (9.8%)</td><td>3364 (3.3%)</td><td>6705 (6.7%)</td><td>8303 (8.3%)</td></tr>
<tr><td>Hibou</td><td>Tissue slides in total 112.5k estimated</td><td>2.5k (2.2%)</td><td>12k (11%)</td><td colspan="2">35k (31%)</td></tr>
<tr><td>Virchow</td><td>Tissue slides</td><td>6.1%</td><td>25%</td><td>3.5%</td><td>3.2%</td></tr>
<tr><td>Phikon, Kaiko (TCGA)</td><td>Patients</td><td>1089 (9.7%)</td><td>979 (8.8%)</td><td>443 (4.0%)</td><td>633 (5.7%)</td></tr>
<tr><td>CTransPath</td><td>Patients</td><td>1089 (8.4%)</td><td>979 (7.5%)</td><td>443 (3.4%)</td><td>1533 (11.7%)</td></tr>
<tr><td>Virchow2</td><td>Tissue slides</td><td>~ 4%</td><td>~ 8%</td><td>~ 2%</td><td>~ 7%</td></tr>
<tr><td>Panakeia</td><td>Patients</td><td>0</td><td>4500 (82%)</td><td>0</td><td>1000 (18%)</td></tr>
</table>

No information available for H-optimus-0, PLIP, BiomedCLIP and DinoSSLPath.

Supplementary methods

Description of foundation models

CTransPath, introduced by Wang et al. in December 2021, is the pioneering Transformer-based unsupervised feature extractor for histopathological images. It integrates a convolutional neural network with a multi-scale Swin Transformer architecture, trained on 15 million patches from 32 cancer subtypes using semantically-relevant contrastive learning, a framework introduced in the same paper based on MoCo v3 [1]. The model was evaluated across multiple tasks, including patch retrieval, patch classification, whole-slide image (WSI) classification, mitosis detection, and colorectal adenocarcinoma gland segmentation [2].

DinoSSLPath, published by Kang et al. in December 2022, employs the DINOv1 framework for SSL [3,4]. DinoSSLPath was pre-trained on 36,666 WSIs, combining TCGA and an internally collected dataset (TULIP). The model uses pathology-specific augmentation techniques, including stain normalization and multi-magnification pretraining at 20× and 40×. DinoSSLPath has been benchmarked on classification tasks and one nuclei instance segmentation task, showing improvements in both label efficiency and dense prediction tasks compared to ImageNet-pretrained baselines.

BiomedCLIP, developed by Zhang et al. in March 2023, is a biomedical vision-language foundation model pretrained on PMC-15M, a dataset of 15 million image-text pairs from PubMed Central [5]. It employs a domain-adapted CLIP framework with PubMedBERT as the text encoder and a ViT-based image encoder. BiomedCLIP excels in cross-modal retrieval, zero-shot classification, and medical visual question answering, achieving state-of-the-art results on diverse biomedical datasets.

Phikon, published by Filiot et al. in July 2023, employs the iBOT framework (image BERT pre-training with Online Tokenizer) for SSL, using MIM and self-distillation [6,7]. The architecture is a ViT-Base model with 80 million parameters, trained on 6,093 WSIs from 16 cancer types, comprising 43 million patches. Phikon was assessed on tile-level and slide-level tasks for subtype, genomic alteration, and overall survival prediction [8].

CONCH, released by Lu et al. in July 2023, is a vision-language model based on CoCa [9], which pretrains an image-text encoder-decoder model using contrastive and captioning losses. For this analysis, only the image encoder was considered. It was pretrained on 16 million image tiles from 21,442 WSIs covering over 350 cancer subtypes. Unlike CTransPath and Phikon, CONCH was trained on proprietary datasets rather than public ones like TCGA and PAIP. The vision-language model is trained by seeking to align image and text modalities in the model's representation space and by predicting the caption corresponding to an image. For this vision-language pretraining, over 1.1 million image-text pairs were used mainly taken from publicly available research articles. Its performance was evaluated on various subtyping, tissue classification, and grading tasks [10].

PLIP, introduced by Huang et al. in August 2023, is a multimodal vision-language foundation model developed for pathology image analysis [11]. Trained on 208,414 pathology image-text pairs from OpenPath—a dataset curated from medical Twitter and other public sources—PLIP

employs contrastive learning to align image and text embeddings. The model demonstrates state-of-the-art performance on zero-shot and few-shot classification tasks and also supports image and text-based retrieval, making it a versatile tool for pathology research and education.

UNI, introduced by Chen et al. in August 2023, is notable for being the first model trained on over 100,000 slides. It utilizes DINOv2 for pretraining, incorporating MIM and self-distillation [12]. The training dataset, Mass-100K, was collected from Massachusetts General Hospital and Brigham and Women's Hospital and consists of over 100 million tissue patches from 20 major tissue types. UNI was tested on the challenging 43-class OncoTree cancer type classification and 108-class OncoTree code classification tasks [13].

Virchow, introduced by Vorontsov et al. in September 2023, stands out as the model trained on the largest dataset to date, with 1.5 million slides from Memorial Sloan Kettering Cancer Center. The model employs DINOv2 for pretraining and features a ViT-Huge architecture with 632 million parameters. Unique to Virchow, the final tile embedding is created using both class tokens and mean patch tokens, doubling the embedding dimension to 2560. It was evaluated on tissue classification and biomarker prediction tasks [14].

Kaiko.ai released a series of pretrained foundation model based on ViT and DINO/DINOv2 [4]. Among these, the ViT-L14 model, trained with DINOv2, was tested in this study. Interestingly, in their own tests, the ViT-B8 trained on DINO performed better on some of the test sets despite using the older SSL method and the smaller ViT-Base architecture. This was attributed to the reduced patch size of eight in comparison to 14 of the ViT-Large. Unlike other recently published foundation models, Kaiko.ai's models were trained on a relatively modest dataset of 29,000 WSIs from TCGA. The performance of these models was assessed on tissue classification tasks using five different datasets [15].

Prov-GigaPath, published by Xu et al. in May 2024, employs a two-stage pretraining approach. Initially, the tile encoder, a ViT-Giant model, is pretrained using DINOv2. Subsequently, a slide encoder contextualizes each tile on the WSI using a LongNet model [16]. Prov-GigaPath was trained on 1.3 billion patches from 170,000 WSIs, sourced from 28 cancer centers within the Providence health network. These WSIs represent over 30,000 patients and encompass 31 major tissue types. Prov-GigaPath was evaluated on mutation prediction using 18 pan-cancer biomarkers and on cancer subtyping tasks, utilizing both TCGA and Providence datasets [17].

PRISM, developed by Shaikovski et al. in May 2024, is a multimodal slide-level foundation model trained on 587,000 WSIs and 195,000 paired clinical reports [18]. Built on Virchow tile embeddings, Prism employs a Perceiver network and BioGPT decoder for zero-shot classification, biomarker prediction, and clinical report generation, achieving state-of-the-art results in low-data scenarios.

Hibou, developed by Nechaev et al. and released in June 2024, includes two versions: Hibou-B and Hibou-L. Hibou-B utilizes a ViT-Base architecture with 86 million parameters and was trained on 510 million tiles. Hibou-L employs a ViT-L architecture trained on 1.2 billion tiles. The training data includes 936,441 H&E and 202,464 non-H&E stained slides from 306,400 individual cases including veterinary biopsies and cytology slides. The performance of the Hibou models was assessed using six datasets for patch-level benchmarks, including tasks such as tissue classification, detection of tumor-infiltrating lymphocytes, and mutation

prediction. Additionally, three datasets were used for slide-level benchmarks, focusing on tissue classification [19].

H-optimus-0 was released on GitHub in July 2024 by Saillard et al. It was trained on a proprietary dataset comprising over 500,000 WSIs, from which hundreds of millions of tiles were extracted. Notably, H-optimus-0 features the highest number of WSIs per patient, with an average of 1.5 slides per patient, compared to other models in this study. For instance, Hibou has 3.7 slides per patient, Prov-GigaPath has 5.7 slides per patient, and Virchow has 12.4 slides per patient. H-optimus-0 employs a ViT-Giant architecture with a patch size of 14 and four registers [20]. The model was evaluated on tile-level tissue classification tasks and slide-level biomarker or metastasis prediction tasks [21].

Virchow2, introduced by Zimmermann et al. in August 2024, expands on the Virchow foundation model by scaling its dataset to 3.1 million WSIs from 225,401 patients, sourced from globally diverse institutions, with mixed magnifications and diverse stains [22]. The model employs a ViT-H/14 architecture with 632 million parameters, trained using domain-specific modifications to the DINOv2 framework, including extended-context translation and KDE regularization. Virchow2 demonstrated state-of-the-art performance on 12 tile-level tasks, significantly improving weighted F1 scores on in-domain and out-of-domain benchmarks compared to its predecessor and other foundation models.

MADELEINE, presented by Jaume et al. in August 2024, leverages multistain pretraining to create slide-level embeddings using a Vision Transformer with multihead attention [23]. Trained on 4,211 breast cancer and 12,070 kidney WSIs with various stains, Madeleine demonstrated robust performance across 21 tasks, including morphological subtyping, molecular prediction, and survival analysis.

CHIEF, introduced by Wang et al. in September 2024, is a general-purpose pathology model trained on 60,530 WSIs across 19 anatomical sites using self-supervised tile-level pretraining and weakly supervised slide-level pretraining [24]. CHIEF demonstrated superior generalizability for cancer detection, tumor origin identification, and biomarker prediction.

The proprietary Panakeia models include two cancer-specific ViT-S for CRC and BRCA. The CRC model was trained on 1,000 slides, generating 4.5 million patches, while the Breast model used 5,000 slides, generating 13 million patches.

The collection of histopathology foundation models analyzed in this study is not exhaustive. Additional models published in the past year include BEPH [25], PLUTO [26], RudolfV [27], PathoDuet [28], and models by Campanella et al. [29]. However, these models are either not publicly accessible (PLUTO, RudolfV, Campanella et al.) or have been trained exclusively on TCGA data using the ViT-Base architecture, which renders them less competitive compared to the more recent models evaluated in this study. Furthermore, the publications associated with these models do not offer comparisons with the latest foundation models.

Comparison of foundation models

In the case of Prov-GigaPath, Xu et al. introduced a slide encoder aimed at analyzing global patterns in WSIs. Previous benchmarking efforts and comparisons by the authors of other foundation models did not evaluate Prov-GigaPath using both tile and slide encoders [19,21,30]. Consequently, we deemed it beneficial to include both versions in this benchmarking study. The results indicate that incorporating the slide encoder does not enhance the performance of the Prov-GigaPath model within a pipeline like STAMP [31]. This is likely because the aggregator model is capable of comprehending slide-level patterns as effectively as the Long-Net model. Thus, it appears feasible to use only the tile encoder in a setup like ours. Similar results were observed for other slide encoders compared to their tile-level counterparts. Using encoded tile embeddings is not beneficial compared to the original tile embeddings; if anything, performance deteriorates. This is likely due to the loss of information, as the encoded embeddings are smaller than the original tile embeddings (**Figure 2G, add. Figure 1A**).

For Virchow, Vorontsov et al. recommended utilizing both the class token (CLS) and the mean patch token (MPT) to create the final tile embedding [14]. This approach doubles the memory requirements for the feature vectors but does not improve performance in our setup. Therefore, it is sufficient to use only the class tokens for Virchow, as is standard practice with the other foundation models in this study. For Virchow2, the same option of using both class tokens and mean patch tokens is available. However, in both cases, we show only the class tokens in the main results, with all versions included for completeness in **Figure S1** and **S10**, and a direct comparison of CLS vs. CLS+MPT for Virchow/Virchow2 in **Add. Fig. 1B**.

CONCH demonstrates exceptional performance despite its relatively modest size as a ViT-Base model. Notably, the vision encoder underwent pretraining on 21,000 WSIs, followed by training the foundation model on over 1.1 million image-text pairs, thus leveraging extensive high-quality training data. Given that the WSIs used for both CONCH and UNI originate from the same source, it is unlikely that there is a qualitative difference between them. The fact that CONCH outperforms UNI, despite being trained on fewer WSIs and utilizing a smaller architecture, underscores the effectiveness of the vision-language approach. These findings suggest that future advancements in histopathological feature extraction may benefit more from the strategic combination of modalities and the utilization of high-quality data rather than merely scaling up model size and data quantity.

Model Ensembles

Combining the prediction scores of different models and concatenating their feature vectors yielded modest performance improvements compared to the best individual models. Heatmap analyses revealed that different models focus on distinct tissue regions and interpret WSIs differently, suggesting potential benefits in leveraging the strengths of multiple foundation models. The approaches explored in this study, however, were relatively rudimentary. Concatenating feature vectors likely introduced redundancy and created excessively long feature vectors, thereby increasing the risk of overfitting, as per Bellman's curse of dimensionality [32]. This might explain why combining the four best feature vectors resulted in inferior performance compared to CONCH alone. Therefore, it would be interesting to find

ways of merging the models without increasing the feature space. To enhance model combination strategies without expanding the feature space, future research could explore dimensionality reduction techniques or the integration of foundation models into a unified framework using merging strategies [33].

Ablation studies

The challenge of overfitting in machine learning models can often be mitigated by increasing the size of the training cohort. Exclusively for this experiment, we leveraged the DACHS dataset for downstream training, which enabled us to utilize up to 1700 patients across six different tasks. We conducted a 5-fold cross-validation on DACHS and evaluated the models on 11 tasks derived from the TCGA and CPTAC datasets. It is worth noting that models such as CTransPath, Phikon, and Kaiko might possess an inherent advantage in this experimental setup due to their pretraining on TCGA data. Our experiments involved varying the training cohort sizes (100, 200, 400, 850, and 1700 patients) to investigate whether larger embedding vectors yield improved performance with larger training cohorts. We correlated the embedding dimension of each foundation model with the mean AUROC across all tasks and five folds. Contrary to our initial hypothesis, the size of the model's embedding vectors did not consistently influence performance in a straightforward manner. Specifically, smaller virchow-class vectors (1280 dimensions) underperformed compared to virchow vectors (2560 dimensions) with a smaller number of patients. However, this performance disparity diminished when the models were trained on cohorts of 850 or 1700 patients. Similarly, Prov-GigaPath-Slide vectors (768 dimensions) performed worse with smaller patient counts compared to Prov-GigaPath vectors (1536 dimensions), but their performance converged when the full patient cohort was used. A noteworthy observation is that the CONCH model exhibited superior performance with limited training data compared to other models. However, this advantage dissipated as the size of the training cohort increased (**Add. Figure 2**).

Data diversity in foundation models

An obvious difference between the foundation models lies in the composition of their training data (Table S6). For instance, Virchow's training data consisted of 25% breast tissue, 18.4% skin, and only 6.1% lung. In contrast, UNI's training data predominantly comprised heart and lung tissues, with less than half as many skin and breast cases. Prov-GigaPath's dataset was 45% lung tissue slides, 30% bowel tissue, and only 2.76% breast tissue. Lastly, CONCH placed greater emphasis on GI and lung tissues, with approximately half as much weight given to breast tissue. Given this variability, Virchow's underperformance in lung cases compared to UNI and Prov-GigaPath may reflect the relatively small proportion of lung cases in its training data. However, the fact that CTransPath outperformed Phikon only in BRCA tasks, despite both being trained on the same BRCA cases from TCGA (as PAIP lacks breast tissue), is contrary to this logic. Overall, there is a moderate correlation ($r = 0.41$) between the number of WSIs of a specific tissue type in the pretraining data and relative performance in downstream tasks involving the same tissue type compared to the average performance of all models in the same tasks (Fig S5). While this correlation is not strong, it underscores the importance of considering tissue type diversity when benchmarking foundation models for histopathology.

Due to relying on the STAMP protocol, it was not feasible to include regression or tissue segmentation tasks, which were often part of the original studies for the foundation models. Additionally, expanding the analysis to include more cancer types would be beneficial, as there are noticeable differences in model performance across various cancers. However, given that CONCH consistently performs best across all analyzed cancer types, it is likely a strong candidate for other tasks as well.

Supplementary Figures

Supplementary Figure 1: Diversity of pretraining datasets

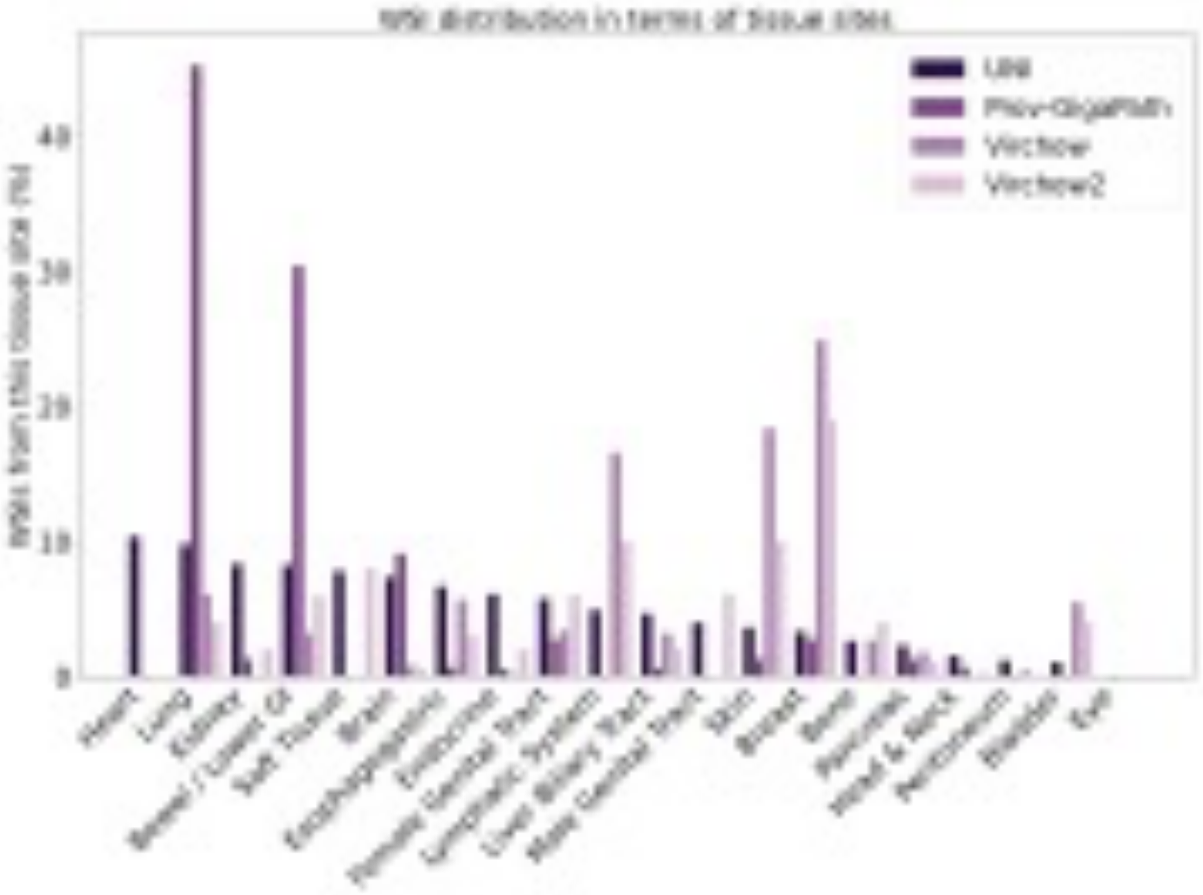

Relative number of slides per anatomic tissue site in the pretraining datasets of four foundation models. For Virchow and Virchow2, upper GI and stomach were grouped into the esophagogastric category, while cervix (Virchow2 only), endometrium, and ovary were combined into the female genital tract. Prostate and testis were combined into the male genital tract for Virchow2. Omentum was considered part of Peritoneum, Bone Marrow under Bone, Thyroid under Endocrine. For Prov-GigaPath, ovary/fallopian tube and uterus were grouped as the female genital tract, and liver with the biliary tract. Data adjustments resulted in Virchow displaying 17 tissue types in 15 categories, Prov-GigaPath with 15 tissue types in 13 categories, and Virchow2 with >175 tissue types in 18 categories.

Supplementary Figure 2: Datasets overview

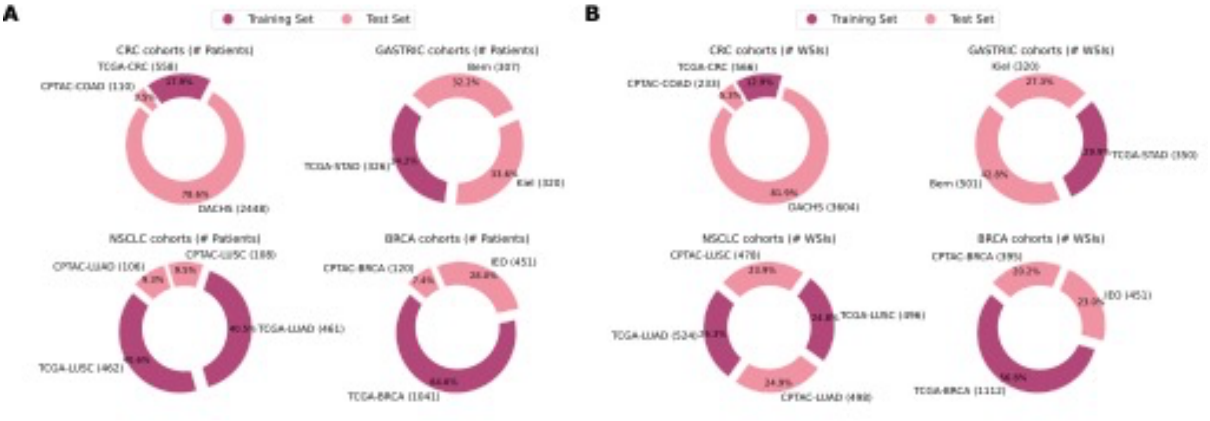

Composition of all cohorts used in this study and the size comparison of training and test sets for each cancer type. Number of patients (**A**) and number of WSIs (**B**) in each cohort. Training was conducted using the TCGA-CRC, TCGA-STAD, TCGA-LUAD, TCGA-LUSC, and TCGA-BRCA cohorts, with TCGA-BRCA being the largest training cohort. Testing was performed using the CPTAC-COAD, CPTAC-LUAD, CPTAC-LUSC, CPTAC-BRCA, DACHS, Bern, Kiel, and IEO cohorts, with DACHS being the largest testing cohort. In total, 6,818 patients and 9,528 slides were used in this study.

Supplementary Figure 3: Comparison of slide encoder models and alternative versions of Virchow and Virchow2

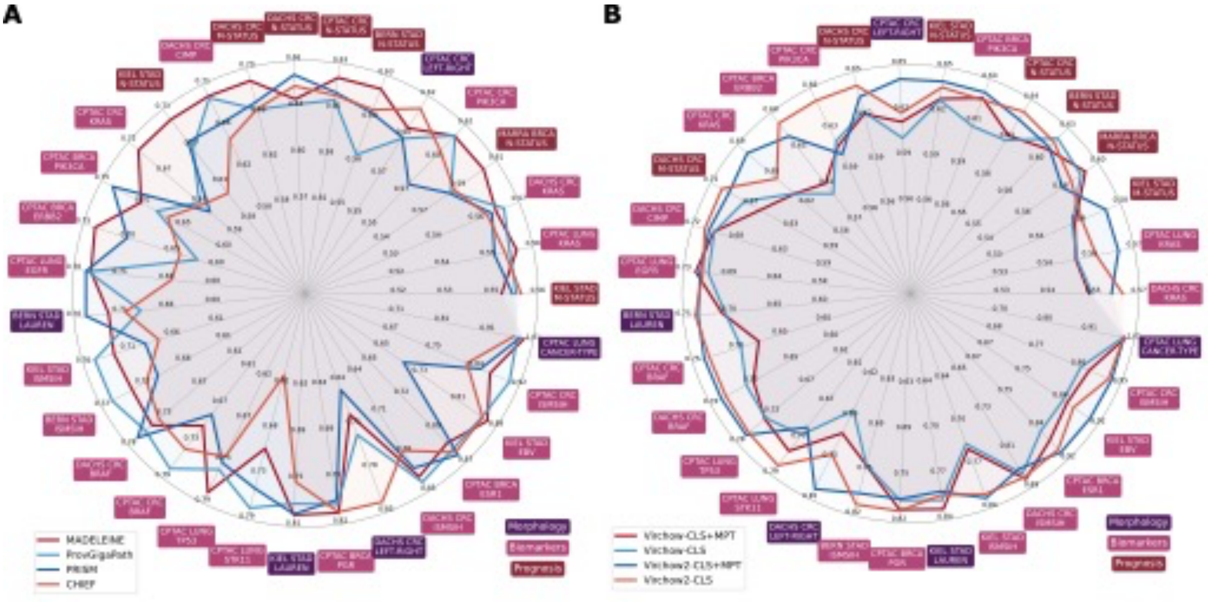

A, Average AUROC scores of slide encoders MADELEINE, Prov-GigaPath, PRISM, and CHIEF across all 31 tasks in the benchmark. **B**, Comparison of two versions of Virchow and Virchow2 (class tokens vs. class tokens + mean patch tokens).

Supplementary Figure 4: Experiments with increased downstream training dataset sizes using DACHS as training cohort

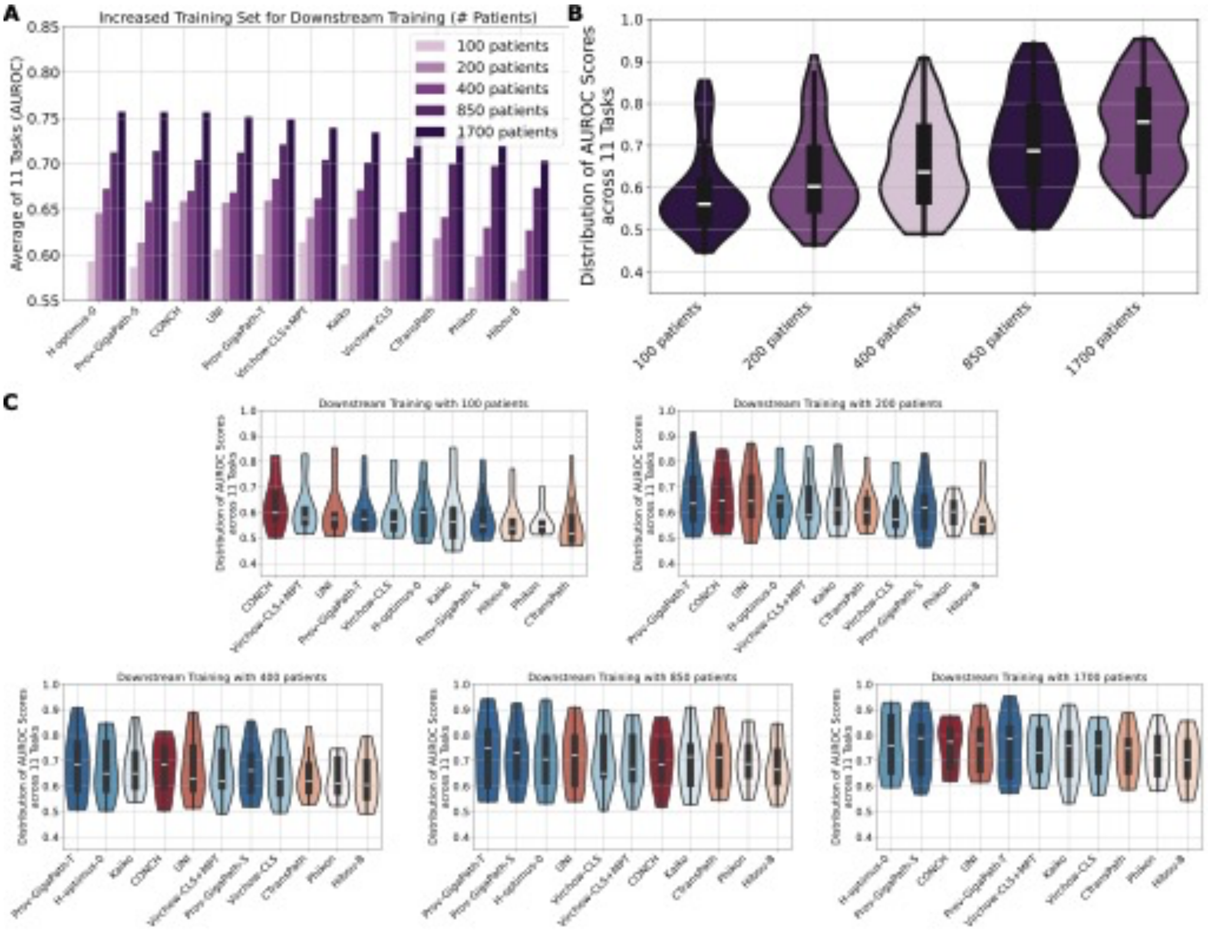

A, Average AUROC across five folds on 11 tasks for models trained on a downstream training dataset with 100, 200, 400, 850, or 1700 patients. **B**, Distribution of AUROC scores from all foundation models grouped by downstream training dataset size. **C**, Distribution of AUROC scores for each foundation model individually. For Prov-GigaPath-T, the tile embeddings were used, for Prov-GigaPath-S, the slide encoder was also included. Virchow-CLS only contained the class tokens, Virchow is the version recommended by the authors. Patients were randomly selected from the DACHS cohort, ensuring ground truth was defined for all analyzed tasks. The task "M-Status," was excluded due to insufficient patient numbers. The models were deployed using the CPTAC-CRC cohort and, unlike other experiments, also included the TCGA-CRC cohort. Consequently, Kaiko, CTransPath, and Phikon models might have an advantage as they had prior exposure to TCGA data during pretraining. Violin plots show kernel density estimates of AUROC scores, truncated at the observed range. The inner box marks the median and interquartile range (25th–75th percentiles), with whiskers extending to the most extreme values within 1.5×IQR.

References

1. Chen, X., Xie, S. & He, K. An empirical study of training self-supervised Vision Transformers. *ICCV* 9620–9629 (2021).
2. Wang, X. *et al.* Transformer-based unsupervised contrastive learning for histopathological image classification. *Med. Image Anal.* **81**, 102559 (2022).
3. Kang, M., Song, H., Park, S., Yoo, D. & Pereira, S. Benchmarking self-supervised learning on diverse pathology datasets. in *2023 IEEE/CVF Conference on Computer Vision and Pattern Recognition (CVPR)* (IEEE, 2023). doi:10.1109/cvpr52729.2023.00326.
4. Caron, M., Touvron, H., Misra, I. & Jégou, H. Emerging properties in self-supervised vision transformers. *Proceedings of the* (2021).
5. Zhang, S. *et al.* BiomedCLIP: a multimodal biomedical foundation model pretrained from fifteen million scientific image-text pairs. *arXiv [cs.CV]* (2023).
6. Bao, H., Dong, L., Piao, S. & Wei, F. BEiT: BERT Pre-Training of Image Transformers. *arXiv [cs.CV]* (2021).
7. Zhou, J. *et al.* iBOT: Image BERT Pre-Training with Online Tokenizer. *arXiv [cs.CV]* (2021).
8. Filiot, A. *et al.* Scaling self-Supervised Learning for histopathology with Masked Image Modeling. *bioRxiv* (2023) doi:10.1101/2023.07.21.23292757.
9. Yu, J. *et al.* CoCa: Contrastive Captioners are Image-Text Foundation Models. *arXiv [cs.CV]* (2022).
10. Lu, M. Y. *et al.* A visual-language foundation model for computational pathology. *Nat. Med.* **30**, 863–874 (2024).
11. Huang, Z., Bianchi, F., Yuksekgonul, M., Montine, T. J. & Zou, J. A visual-language foundation model for pathology image analysis using medical Twitter. *Nat. Med.* **29**, 2307–2316 (2023).
12. Oquab, M. *et al.* DINOv2: Learning Robust Visual Features without Supervision. *arXiv*

[cs.CV] (2023).

13. Chen, R. J. *et al.* Towards a general-purpose foundation model for computational pathology. *Nat. Med.* **30**, 850–862 (2024).
14. Vorontsov, E., Bozkurt, A., Casson, A. & Shaikovski, G. A foundation model for clinical-grade computational pathology and rare cancers detection. *Nat. Med.* (2024).
15. Ai, K. *et al.* Towards Large-Scale Training of Pathology Foundation Models. *arXiv [cs.CV]* (2024).
16. Ding, J. *et al.* LongNet: Scaling Transformers to 1,000,000,000 Tokens. *arXiv [cs.CL]* (2023).
17. Xu, H. *et al.* A whole-slide foundation model for digital pathology from real-world data. *Nature* **630**, 181–188 (2024).
18. Shaikovski, G. *et al.* PRISM: A multi-modal generative foundation model for slide-level histopathology. *arXiv [eess.IV]* (2024).
19. Nechaev, D., Pchelnikov, A. & Ivanova, E. Hibou: A Family of Foundational Vision Transformers for Pathology. *arXiv [eess.IV]* (2024).
20. Darcet, T., Oquab, M., Mairal, J. & Bojanowski, P. Vision Transformers Need Registers. *arXiv [cs.CV]* (2023).
21. Saillard, C. *et al. H-Optimus-0.* (2024).
22. Zimmermann, E. *et al.* Virchow2: Scaling self-supervised mixed magnification models in pathology. *arXiv [cs.CV]* (2024).
23. Jaume, G. *et al.* Multistain pretraining for slide representation learning in pathology. *arXiv [eess.IV]* (2024).
24. Wang, X. *et al.* A pathology foundation model for cancer diagnosis and prognosis prediction. *Nature* **634**, 970–978 (2024).
25. Yang, Z. *et al.* A foundation model for generalizable cancer diagnosis and survival prediction from histopathological images. *bioRxiv* 2024.05.16.594499 (2024) doi:10.1101/2024.05.16.594499.
26. Juyal, D. *et al.* PLUTO: Pathology-Universal Transformer. *arXiv [eess.IV]* (2024).

27. Dippel, J. *et al.* RudolfV: A Foundation Model by Pathologists for Pathologists. *arXiv [eess.IV]* (2024).
28. Hua, S., Yan, F., Shen, T. & Zhang, X. PathoDuet: Foundation Models for Pathological Slide Analysis of H&E and IHC Stains. *arXiv [cs.CV]* (2023).
29. Campanella, G. *et al.* Computational Pathology at Health System Scale -- Self-Supervised Foundation Models from Three Billion Images. *arXiv [cs.CV]* (2023).
30. Campanella, G. *et al.* A Clinical Benchmark of Public Self-Supervised Pathology Foundation Models. *arXiv [eess.IV]* (2024).
31. El Nahhas, O. S. M. *et al.* From Whole-slide Image to Biomarker Prediction: A Protocol for End-to-End Deep Learning in Computational Pathology. *arXiv [cs.CV]* (2023).
32. Bellman, R. *Dynamic Programming*. (Princeton University Press, 1957).
33. Ainsworth, S. K., Hayase, J. & Srinivasa, S. Git Re-Basin: Merging Models modulo Permutation Symmetries. *arXiv [cs.LG]* (2022).
