# Supplementary material for: Benchmarking foundation models as feature extractors for weakly supervised computational pathology
Source: Nat Biomed Eng. 2025 Oct 1;10(6):1113–23. doi: 10.1038/s41551-025-01516-3 (PMC13279263; doi:10.1038/s41551-025-01516-3)
Supplement: Supplementary file 2 — Reporting Summary [file 41551_2025_1516_MOESM2_ESM.pdf]

nature portfolio

Corresponding author(s): Jakob Nikolas Kather

Last updated by author(s): Jul 1, 2025

Reporting Summary

Nature Portfolio wishes to improve the reproducibility of the work that we publish. This form provides structure for consistency and transparency in reporting. For further information on Nature Portfolio policies, see our Editorial Policies and the Editorial Policy Checklist.

Statistics

For all statistical analyses, confirm that the following items are present in the figure legend, table legend, main text, or Methods section.

n/a	Confirmed	
☐	☒	The exact sample size (*n*) for each experimental group/condition, given as a discrete number and unit of measurement
☐	☒	A statement on whether measurements were taken from distinct samples or whether the same sample was measured repeatedly
☐	☒	The statistical test(s) used AND whether they are one- or two-sided<br>*Only common tests should be described solely by name; describe more complex techniques in the Methods section.*
☒	☐	A description of all covariates tested
☐	☒	A description of any assumptions or corrections, such as tests of normality and adjustment for multiple comparisons
☐	☒	A full description of the statistical parameters including central tendency (e.g. means) or other basic estimates (e.g. regression coefficient) AND variation (e.g. standard deviation) or associated estimates of uncertainty (e.g. confidence intervals)
☐	☒	For null hypothesis testing, the test statistic (e.g. *F*, *t*, *r*) with confidence intervals, effect sizes, degrees of freedom and *P* value noted<br>*Give P values as exact values whenever suitable.*
☒	☐	For Bayesian analysis, information on the choice of priors and Markov chain Monte Carlo settings
☒	☐	For hierarchical and complex designs, identification of the appropriate level for tests and full reporting of outcomes
☐	☒	Estimates of effect sizes (e.g. Cohen's *d*, Pearson's *r*), indicating how they were calculated

Our web collection on statistics for biologists contains articles on many of the points above.

Software and code

Policy information about availability of computer code

Data collection	No software was used for the collection of data.
Data analysis	All source codes are available under an open-source license on Github. The STAMP pipeline is found at https://github.com/KatherLab/STAMP-Benchmark.<br>We used 19 pretrained models for tile-level and slide-level feature extraction. To ensure reproducibility, we record the full SHA-256 hash of each model's weight file. CTransPath has SHA-256: 7c998680060c8743551a412583fac689db43cec07053b72dfec6dcd810113539. DinoSSLPath was obtained from Lunit's official GitHub release with SHA-256: 75389172cd77cef7dd511f3077f38e8133d6c7cdf33b2f7c23aa8534094ef6ca. Phikon (owkin/phikon) has SHA-256: d01642bfeb683c6b039622eca273e0a8e4208065a4595b63edba178c1c4cfa68). UNI encoder (MahmoodLab/UNI) has SHA-256: 56ef09b44a25dc5c7eedc55551b3d47bcd17659a7a33837cf9abc9ec4e2ffb40). For Virchow (paige-ai/Virchow) and Virchow2 (paige-ai/Virchow2): SHA-256: 3416891b37a2349a2d9ce7ecf00b64a6277011b41351436c1ceb0abb80805408 and 8d6cea947eb2418c3b0dff48cfb9b238e47744ab0dfca21b2b0637b140769b4b). Kaiko ViT-L/14 (kaiko-ai/towards_large_pathology_fms): SHA-256: 564c84d185369cb48ef78abbc020c89bfc725daf56460ab7ead54be573a37d98. Prov-GigaPath tile encoder : SHA-256: 877947214318afa9e011754b74bbc3894a1f480a253afc7bc8045b8321dedd63. Hibou-B and Hibou-L (histai/hibou-b and histai/hibou-l): SHA-256: 2121db3cac83bc9abf13a458a37b0740e2ce725609ff8d4b8e6b6c56c30c3ab6 and a63d1699448143ceccf2b7e1c4bf35b995c51f86ef8a6efb471af2eee210b79c. H-Optimus-0 (bioptimus/H-optimus-0): SHA-256: bf47b8467403079798661601b6ab06533be789da48d3f8b510aaefe2fbc55cd3. For vision-language models, we used PLIP (vinid/plip, SHA-256: 98a7f8d2a1f4a8fc8f6dedb3a16ff7efbe02a7ef67c93904c80bca9767c69630), BiomedCLIP (microsoft/BiomedCLIP-PubMedBERT_256-vit_base_patch16_224, SHA-256: 52cc993c5c5ff962bd0c60931874bc001e7e9b41666a385530f4a036294576be) and CONCH (from GitHub, SHA-256: 40a9644b9ba0e83a74576e0a5e5f7313599fa9c9cdaf3c20f8a3e271b0e9ae7c). For slide-level encoders, we used GigaPath (SHA-256: 04194a4c393e6a713b144356791c0d2e27ca769ee50c6e111fd6abc1f17ed551), MADELEINE (SHA-256:

34437fe7cf6e1d9b6fb41ef592416ef890dc07c599ca1cc8d1ff00c40ce23496), PRISM (SHA-256: 01a0f7bcfd1559de31794e08a99e9fe4f9bd758c3b9f1482fe9cb3f5e1e7c5e1), and CHIEF (SHA-256: 6a46d200b32a65e5ce4774611b889b5f1bbf7a39f9111321a2a1b5dbdb9996b8). Panakeia is proprietary and not publicly distributed. Feature extraction and inference were implemented in Python 3.11.8 using PyTorch 2.3.1 (CUDA 12.1), torchvision 0.18.1, timm 1.0.3, transformers 4.31.0, open_clip_torch 2.23.0, uni 0.1.0, Pillow 10.3.0, and NumPy 1.26.4.

For manuscripts utilizing custom algorithms or software that are central to the research but not yet described in published literature, software must be made available to editors and reviewers. We strongly encourage code deposition in a community repository (e.g. GitHub). See the Nature Portfolio guidelines for submitting code & software for further information.

Data

Policy information about availability of data

All manuscripts must include a data availability statement. This statement should provide the following information, where applicable:

- Accession codes, unique identifiers, or web links for publicly available datasets
- A description of any restrictions on data availability
- For clinical datasets or third party data, please ensure that the statement adheres to our policy

The slides for TCGA are available at https://portal.gdc.cancer.gov/. The slides for CPTAC are available at https://proteomics.cancer.gov/data-portal. The molecular data for TCGA and CPTAC are available at https://www.cbioportal.org/. The slides and biomarker data for DACHS were generated for prior studies56–58 with restricted access. Biomarker data for DACHS are available by requesting Authorized Access to the phs001078 study [https://www.ncbi.nlm.nih.gov/projects/gap/cgi-bin/study.cgi?study_id=phs001113.v1.p1]. Applications for access to DACHS biomarker data are reserved for Senior Investigators and NIH Investigators as defined in https://dbgap.ncbi.nlm.nih.gov/aa/wga.cgi, and upon successful application grants access to the data for 1 year with the option to renew access. The slides for DACHS can only be requested directly through the DACHS principal investigators. The contact details are listed at http://dachs.dkfz.org/dachs/kontakt.html. The Kiel cohort is available from the Department of Pathology, Christian Albrechts University of Kiel, Kiel, Germany, upon reasonable request (https://www.medizin.uni-kiel.de/en/institutes-departments/institutes-of-clinical-theory/department-of-pathology). The Bern cohort is proprietary and cannot be shared at the individual patient level. It is archived at the Institute of Pathology, University of Bern, and can be requested in reference to the original study: Dislich B, Blaser N, Berger MD, et al. Histopathology. 2020;76(5):740–747. The IEO cohort is held by the European Institute of Oncology, Milan. Data requests will be evaluated on a case-by-case basis in accordance with institutional policies and privacy regulations. The data generated in this study for the creation of the figures are provided in the Source Data file. Source data are provided with this paper.

Research involving human participants, their data, or biological material

Policy information about studies with human participants or human data. See also policy information about sex, gender (identity/presentation), and sexual orientation and race, ethnicity and racism.

Reporting on sex and gender	Results are irrespective of sex or gender.
Reporting on race, ethnicity, or other socially relevant groupings	The country of origin for cohort TCGA is the US, for cohort CPTAC is the US, for cohort DACHS and cohort Kiel is Germany, for cohort Bern is Switzerland and for cohort IEO is Italy. No information about race was available.
Population characteristics	No data on population characteristics was collected and used in this study.
Recruitment	All studies contain samples which were collected with the intent of performing translational research.
Ethics oversight	This study was carried out in accordance with the Declaration of Helsinki. The Clinical Proteomic Tumor Analysis Consortium (CPTAC) and TCGA did not require formal ethics approval for a retrospective study of anonymised samples. The analysis of the testing cohort DACHS (an epidemiological study which is led by the German Cancer Research Center, DKFZ, Heidelberg, Germany) was approved by the ethics committee of the Medical Faculty, University of Heidelberg under 310/2001.

Note that full information on the approval of the study protocol must also be provided in the manuscript.

Field-specific reporting

Please select the one below that is the best fit for your research. If you are not sure, read the appropriate sections before making your selection.

☒ Life sciences ☐ Behavioural & social sciences ☐ Ecological, evolutionary & environmental sciences

For a reference copy of the document with all sections, see nature.com/documents/nr-reporting-summary-flat.pdf

Life sciences study design

All studies must disclose on these points even when the disclosure is negative.

Sample size	No explicit sample-size calculation was performed. Recent studies in computational pathology by Foersch et al. (Nat Med, 2023) and Wagner et al. (Cancer Cell, 2023) showed successful biomarker predictions with cohorts having several hundred patients, with larger cohorts yielding better results. Consequently, we collected datasets which satisfied this volume range of patients, having several hundreds, or thousands of patients available for retrospective analysis. Moreover, a recent Nature Protocols Paper by El Nahhas et al. provided rough guidelines for an estimation of sample sizes for computational pathology (https://www.nature.com/articles/s41596-024-01047-2).

Data exclusions	In all experiments, data samples were excluded when the microns-per-pixel information was not available in the metadata of the whole-slide image, or if the biomarker to be predicted was not available for the sample.
Replication	Random seeds set in the code, patient splits for training and testing models were saved, and the code version was managed through GitHub for reproducibility.
Randomization	Samples were stochastically allocated into different groups, where the model was trained, validated and tested on samples from different patients and different hospitals. More details in the methods and accompanying code.
Blinding	This study was conducted retrospectively. Therefore, investigators were not blinded to allocation during experiments or outcome assessment. The data was randomly split into training and validation sets for the training cohort (TCGA), while all other cohorts were used exclusively for external validation. Given this design, blinding was not applicable.

Reporting for specific materials, systems and methods

We require information from authors about some types of materials, experimental systems and methods used in many studies. Here, indicate whether each material, system or method listed is relevant to your study. If you are not sure if a list item applies to your research, read the appropriate section before selecting a response.

Materials & experimental systems

n/a	Involved in the study	
☒	☐	Antibodies
☒	☐	Eukaryotic cell lines
☒	☐	Palaeontology and archaeology
☒	☐	Animals and other organisms
☒	☐	Clinical data
☒	☐	Dual use research of concern
☒	☐	Plants

Methods

n/a	Involved in the study	
☒	☐	ChIP-seq
☒	☐	Flow cytometry
☒	☐	MRI-based neuroimaging

Plants

Seed stocks	*Report on the source of all seed stocks or other plant material used. If applicable, state the seed stock centre and catalogue number. If plant specimens were collected from the field, describe the collection location, date and sampling procedures.*
Novel plant genotypes	*Describe the methods by which all novel plant genotypes were produced. This includes those generated by transgenic approaches, gene editing, chemical/radiation-based mutagenesis and hybridization. For transgenic lines, describe the transformation method, the number of independent lines analyzed and the generation upon which experiments were performed. For gene-edited lines, describe the editor used, the endogenous sequence targeted for editing, the targeting guide RNA sequence (if applicable) and how the editor was applied.*
Authentication	*Describe any authentication procedures for each seed stock used or novel genotype generated. Describe any experiments used to assess the effect of a mutation and, where applicable, how potential secondary effects (e.g. second site T-DNA insertions, mosiacism, off-target gene editing) were examined.*
