# Supplementary material for: Benchmarking foundation models as feature extractors for weakly supervised computational pathology
Source: Nat Biomed Eng. 2025 Oct 1;10(6):1113–23. doi: 10.1038/s41551-025-01516-3 (PMC13279263; doi:10.1038/s41551-025-01516-3)
Supplement: Supplementary file 3 — Peer Review File [file 41551_2025_1516_MOESM3_ESM.pdf]

nature portfolio

# Peer Review File

# Benchmarking foundation models as feature extractors for weakly-supervised computational pathology

Corresponding Author: Professor Jakob Kather

Version 0:

Decision Letter:

Dear Jakob,

Thank you again for submitting to *Nature Biomedical Engineering* your manuscript, "Benchmarking foundation models as feature extractors for weakly-supervised computational pathology". The manuscript has been seen by two experts, whose reports you will find at the end of this message.

You will see that the reviewers appreciate the aims of the model-benchmarking effort. However, they articulate serious concerns about the usefulness, methodology and completeness of the benchmarking as well as about the interpretation of the performance comparisons, and provide useful suggestions for improvement. We hope that with substantial further effort you can address the criticisms, increase the strength of the evidence, and convince the reviewers of the merits of the study.

When you are ready to resubmit your manuscript, please <a href="Link Redacted">upload</a> the revised files, a point-by-point rebuttal to the comments from all reviewers, the <a href="https://www.nature.com/authors/policies/ReportingSummary.pdf">reporting summary</a>, and a cover letter that explains the main improvements included in the revision and responds to any points highlighted in this decision.

Please follow the following recommendations:

* Clearly highlight any amendments to the text and figures to help the reviewers and editors find and understand the changes (yet keep in mind that excessive marking can hinder readability).

* If you and your co-authors disagree with a criticism, provide the arguments to the reviewer (optionally, indicate the relevant points in the cover letter).

* If a criticism or suggestion is not addressed, please indicate so in the rebuttal to the reviewer comments and explain the reason(s).

* Consider including responses to any criticisms raised by more than one reviewer at the beginning of the rebuttal, in a section addressed to all reviewers.

* The rebuttal should include the reviewer comments in point-by-point format (please note that we provide all reviewers will the reports as they appear at the end of this message).

* Provide the rebuttal to the reviewer comments and the cover letter as separate files.

We expect that you will be able to resubmit the manuscript within 16 weeks of receiving this message. If this is the case, you will be protected against potential scooping. Otherwise, we will be happy to consider a revised manuscript as long as the significance of the work is not compromised by work published elsewhere or accepted for publication at *Nature Biomedical Engineering*.

We hope that you will find the referee reports helpful when revising the work. Please do not hesitate to contact me should you have any questions.

Best wishes,

Pep

__
Pep Pàmies
Chief Editor, <a href="http://www.nature.com/nbme">*Nature Biomedical Engineering*</a>

__________
Reviewer #1 (Report for the authors (Required)):

This paper conducts an evaluation comparing 9 pathology foundation models on 31 classification tasks. Given the abundant availability of pathology FMs, a comprehensive benchmark evaluation could potentially provide valuable insight to aid future progress in digital pathology. Therefore, such studies should be encouraged. That being said, this study in its current form is lacking in many key aspects, making it hard to assess its true significance.

The main conclusion that the authors highlighted is that CONCH attained higher performance over other models. However, this is not surprising at all, given that CONCH was trained using images with text annotations, including cancer type, biomarker, patient status, etc. The whole point of the CLIP-based contrastive learning in CONCH is to imbue the image encoder with key diagnostic information from the caption. Specifically, CONCH was pretrained using the pathology subset of PMC figure-caption pairs from BiomedCLIP. Both CONCH and BiomedCLIP have demonstrated zero-shot capabilities for various pathology tasks, including morphology and biomarker classification.

This would also perfectly explain why CONCH appear to have especially good performance in low-data regime.

Additionally, as the authors observed, a few models were pretrained using TCGA data and thus might have built-in advantages. Given TCGA's wide spread use in pathology studies, PMC pathology images might have a non-trivial overlap with TCGA, which means that CONCH might benefit from the same built-in advantage as well (in addition to contrastive training using caption).

After excluding CONCH, which is the only model that has undergone such training, it appears that on average, most models perform basically the same (e.g., Fig S4). Overall, it's hard to derive any meaningful insights among the non-CONCH models.

In light of this, the paper seems to be a bit too eager to draw premature conclusions. E.g., the authors concluded that "CONCH generally outperforms other foundation models across all task categories, followed by UNI and H-optimus" (Figure 1), but it's hard to see why UNI and H-optimus should be singled out as the next best two models, whether from the Figure itself (e.g., Virchow appears to outperform both UNI and Optimus in morphology tasks; GigaPath seems tied or slightly better than UNI), or from detailed performance break-down in supplementary (e.g., S4).

Compounding the challenge in interpreting the results is the composition of the test set. Some categories have very few instances (e.g., 15 for CRC BRAF MUT), which means that the test score might not be truly representative. Also, the paper claims to test on 31 tasks, each of which comprises a dataset (e.g., DACHES vs CPTAC), a cancer type (e.g., LUAD), and a classification problem (e.g., whether the patient is ERBB2 positive. However, it seems that half of the test tasks came from CPTAC (15 tasks), which had obvious overlap with TCGA, hence undermining the claim that this represents a truly external validation. E.g., some of the test instances might be present in TCGA training data, or even in the CONCH vision-language pretraining.

Overall, while one would certainly appreciate more clarity, it could be detrimental to future research if unwarranted conclusions were drawn prematurely from inconclusive evidence.

There are also a few questions regarding the training process. A WSI may comprise up to tens of thousands of tiles. As the authors pointed out, all but GigaPath are tile-encoders, which are typically applied to slide-level classification using MIL (e.g., ABMIL). However, the paper only mentioned multi-head self-attention and MLP for aggregation, but didn't specify any further details. How large is each slide in terms of tile number? How is self-attention applied (e.g., what position embedding was used for each tile)? Given the large tile number, wouldn't the method suffer from quadratic growth in compute (which is exactly why most non-GigaPath models used ABMIL or other MIL methods).

This is even more puzzling when it comes to GigaPath. The authors mentioned concatenating slide and tile encoder, but there are many tiles in a slide. Plus, wouldn't the whole point for the slide encoder is to capture info from all the slides (which the authors also seemed to allude to in a side note comparing their dimensions)? Likewise, how does CONCH + GigaPath work, given that CONCH image encoder is tile-level?

The ML setup is also a bit head-scratching. The authors conducted five-fold cross-validation on TCGA training data, applied each of the five models to test set, and then computed the average score for each task. This raises several questions about the training setup.

First, how did the authors pick hyperparameters during each of the five-fold training? Second, why not train a single model that uses all TCGA data? In fact, n-fold CV is often used for hyperparameter selection on the training data (if there is no dev set for this), followed by training on the entire training data using the chosen hyperparameters.

Additionally, Some categories have very few training data (e.g., only 10 instances are available for STAD LAUREN MIXED), and cross-validation would exacerbate the scarcity of training data. It's unclear how the five folds are divided. If it's random split, by sheer coincidence some training run might have close to zero instance for rare categories, thus further magnifying CONCH's advantage given its training based on natural-text annotations.

As a minor note, why did the authors choose to tile the slide by 224 X 224, rather than following each original method's setting? This could have an especially large effect on GigaPath, given that its slide-level encoder was pretrained assuming 256 X 256 tiling, thus learned a position embedding that would be rather different if the tiling was 224 X 224. Likewise, it's also puzzling why the authors would evaluate on H-optimus tile encoder + GigaPath slide encoder, given that H-optimus was tiled as 224 X 224, as the authors used in the experimental setting. Given the mismatched tiling, it's hard to see what is the point for such experiments.

Reviewer #2 (Report for the authors (Required)):

This study evaluates the performance of histopathology foundation models across lung, colorectal, gastric, and breast cancer patient cohorts. The models were tested on morphological classification, biomarker prediction, and prognostic prediction tasks. The authors showed that CONCH generally performs the best compared to other models that only use imaging inputs during training. In addition, the authors ensembled different models and showed that combining model predictions led to minor performance improvements on these tasks. They further explored the influence of data set size and data diversity on fine-tuning these models. Below are my comments.

1. The evaluation tasks presented in the current manuscript are somewhat limited. Only four cancer types were evaluated. The original papers for some of the foundation models under study included comparisons with other published models on tens or more cancer types. Because different foundation models are known to have varying performance across cancer types and diagnostic tasks, the choice of tasks for evaluation can impact the conclusion of the study. The authors could consider conducting a more comprehensive analysis using tasks presented in these original foundation model papers.

2. The best-performing model has an average area under the receiver operating characteristic curve (AUROC) of 0.77 in morphology prediction tasks, which seems surprisingly low, considering that trained pathologists can identify these diagnostic categories with minimal inter-rater disagreement. The authors could discuss potential reasons for this finding.

3. Several prior papers, such as Kather et al. (Nature Cancer 2020) and Fu et al. (Nature Cancer 2020), have systematically analyzed biomarker prediction using hematoxylin and eosin-stained pathology images and showed a wide range of prediction results for different biomarkers. In this manuscript, only a subset of these tasks is selected and presented, and it is unclear how these biomarkers were selected. For example, NTRK mutations are relevant for targeted therapy in stomach cancer, and BRAF and MET mutations are important for treatment decisions in lung adenocarcinoma. However, these genomic alterations were not included in the current analyses. Given the variability of model performance across different tasks, the selection of biomarkers could influence the presented results.

4. The evaluation results from Panakeia are missing from Figure 2F. It is unclear whether this foundation model selected by the authors was assessed in any of the proposed analyses and comparisons with other foundation models.

5. The authors did not include pathology language-image pretraining (PLIP), a well-known vision-language foundation model for pathology image analyses. PLIP is also known to be one of the first vision-language models for digital pathology evaluation. Without its inclusion, only one model under study used a language component.

6. There are several other newer foundation models, such as DinoSSLPath, CHIEF, and Virchow2, that the authors did not include in their analyses. Because several recent papers claimed that these models have even better performance, it would be interesting to see how these newer foundation models compare with those currently being evaluated in their study settings.

7. It would be interesting to compare GPT-4o or other general-purpose multi-modal foundation models with their current results. The authors noted that multi-modal approaches yield significantly improved results. It would be crucial to evaluate the performance of multi-modal methods not specific to pathology when further trained or fine-tuned for pathology evaluation tasks.

8. According to the current results presented by the authors, many high-performing foundation models employed pen marks and other artifacts during diagnostic evaluation. This is concerning because these signals are not directly related to the classification tasks and should not be relied on. It would be helpful to quantify the extent to which pen marks contributed to the prediction results of these models.

9. Although CONCH used a different pretraining approach involving image-caption pairs, it is still possible to investigate the impact of the proportion of slides included in the training dataset on its performance. The current analyses excluded this and many other models in the analyses on how data diversity influences performance.

10. The performance improvement achieved by the proposed ensembling method (1.3% improvement in AUROC) seems

very low. It would be interesting to explore the reasons behind the minimal performance improvement despite combining models with different strengths.

This GitHub link seems to be associated with a paper published in Nature Protocols. This paper used this published tool, although the tool is not specific to this paper.

Version 1:

Decision Letter:

Dear Professor Kather,

Your manuscript "Benchmarking foundation models as feature extractors for weakly-supervised computational pathology" has now been seen by 2 of the previous reviewers. In light of their advice and on the basis of our editorial evaluation, I would be happy to consider a revised version of the manuscript.

Reviewer 1 has some further concerns that should be addressed before we move forward with publication.

As before, when you are ready to resubmit your manuscript, please <a href="Link Redacted">upload</a> the revised files, a point-by-point rebuttal to the comments from all reviewers, and a cover letter that explains the main improvements included in the revision and responds to any points highlighted in this decision.

As a reminder, please follow the following recommendations:

* Please submit your revised manuscript in a word doc or LaTeX format.

* Clearly highlight any amendments to the text and figures to help the reviewers and editors find and understand the changes (yet keep in mind that excessive marking can hinder readability).

* If you and your co-authors disagree with a criticism, provide the arguments to the reviewer (optionally, indicate the relevant points in the cover letter).

* If a criticism or suggestion is not addressed, please indicate so in the rebuttal to the reviewer comments and explain the reason(s).

* Consider including responses to any criticisms raised by more than one reviewer at the beginning of the rebuttal, in a section addressed to all reviewers.

* The rebuttal should include the reviewer comments in point-by-point format (please note that we provide all reviewers will the reports as they appear at the end of this message).

* Provide the rebuttal to the reviewer comments and the cover letter as separate files.

We expect that you will be able to resubmit the manuscript within 8 weeks of receiving message. If this is the case, you will be protected against potential scooping. Otherwise, we will be happy to consider the next version of the manuscript as long as the advance of the work is not compromised by work published elsewhere or accepted for publication at *Nature Biomedical Engineering*.

We look forward to receive a further revised version of the work. Please do not hesitate to contact me should you have any questions.

Best wishes,

Barbara Cheifet
Editor
Nature Biomedical Engineering

---

Reviewer #1 (Report for the authors (Required)):

I want to thank the authors for detailed and substantive responses. It addresses all my major concerns. I'm particularly heartened to see the efforts to preempt train-test contamination. Additional experiments on several newer models also help strengthen the paper significantly.

A couple minor notes:

I'm a bit confused by Fig 2F. The task numbers for each category (e.g., morphology) don't seem to add up to the same number across different models (e.g., 17 for Conch, 16 for Virchow2, 15 for GigaPath, 13 for DinoSSLPath. Did I miss something?

I'm still quite a bit confused by the comparison of slide-level encoder vs tile-level encoder (Fig 2G): "For histopathology slide encoders, we retrieved the encoded tile-level embeddings to make them applicable to our MIL approach. The original tile embeddings consistently outperformed their slide-level counterparts and the performance of the encoded tile embeddings is driven by the quality of the original tile embeddings and not by the slide encoder (Figure 2G)."

It seems that the authors use MIL approach that randomly sample 512 (out of tens of thousands?) tiles and aggregate them as feature vectors. The fact that such a MIL approach can perform strongly suggest that the underlying tasks do not depend much on global patterns spanning the whole slide, but are rather largely dependent on local tile-level patterns. Is that correct? Ofc, this doesn't detract from the value of the study, but merely reflects the predominant characteristics of existing pathology benchmarks. Would be great to see some discussion here.

The authors mentioned in rebuttal: "We appreciate the reviewer's perspective on CONCH's anticipated strength in low-data scenarios, though our findings show that this assumption does not consistently hold (Fig. S6, S2B). CONCH ranks as the second-best model across all reduced downstream dataset experiments with 75, 150, and 300 patients. Similarly, for low-prevalence tasks, CONCH performs comparably to others but does not stand out." I really like these additional analyses that shed light on CONCH's performance on low-data regime that's a bit counterintuitive. However, I don't seem to see them highlighted in the abstract or main text, which still left the impression that CONCH aced in all conditions. Maybe I miss some mention, but think interesting results like these are worth highlighting more prominently, rather than relegated to supplement.

Reviewer #2 (Report for the authors (Required)):

This study evaluates previously published histopathology foundation models using cancer samples, focusing on weakly supervised tasks in cancer diagnosis. It highlights the complementarity of models trained on diverse datasets, demonstrating that combining them enhances predictive accuracy. The findings suggest that pathology machine learning models could be further improved through combinatorial approaches. This revision addressed my previous comments. Thank you.

Reviewer #2 (Remarks on code availability):

The README file contains instructions for using the application.

Version 2:

Decision Letter:

Dear Professor Kather,

Thank you for your revised manuscript, "Benchmarking foundation models as feature extractors for weakly-supervised computational pathology". I am pleased to write that we shall be happy to publish the manuscript in *Nature Biomedical Engineering*.

We will be performing detailed checks on your manuscript, and in due course will send you a checklist detailing our editorial and formatting requirements. You will need to follow these instructions before you upload the final manuscript files.

Please do not hesitate to contact me if you have any questions.

Best wishes,

Barbara Cheifet
Editor
Nature Biomedical Engineering

___________

Version 3:

Decision Letter:

Dear Professor Kather,

I am happy to inform you that your manuscript, "Benchmarking foundation models as feature extractors for weakly-supervised computational pathology", has now been accepted for publication in *Nature Biomedical Engineering*.

Over the next few weeks, the figures will be checked for production quality, the text edited to ensure that it conforms to house style, and the manuscript typeset.

Our Articles are published about 40 days after the acceptance date (we recommend that you inform your institutional press office of this timeframe), and you will be notified of the actual publication date a few days in advance. Articles can be published any working day of the week, and are pushed live shortly after 10 am London time.

**Publishing agreement.** You will be asked to digitally sign a publishing agreement (grant of rights). After the signed publishing agreement has been received, the proofs of the article will be sent to you for review. If you have any queries during the production process, or you cannot meet the requested deadline for returning the proofs, please contact rjsproduction@springernature.com.

*Nature Biomedical Engineering* is a Transformative Journal. Authors may publish their research with us through the traditional subscription access route, or make their paper immediately open access through payment of an article-processing charge. More <a href="https://www.springernature.com/gp/open-research/transformative-journals">information about publication options</a> is available.

**You may need to take specific actions to <a href="https://www.springernature.com/gp/open-research/funding/policy-compliance-faqs">comply</a> with funder and institutional open-access mandates**. If the work described in the accepted manuscript is supported by a funder that requires immediate open access (as outlined, for example, by <a href="https://www.springernature.com/gp/open-research/plan-s-compliance">Plan S</a>) and your manuscript was originally submitted on or after January 1st 2021, then you should select the gold OA route. Authors selecting subscription publication will need to accept our standard licensing terms (including our <a href="https://www.springernature.com/gp/open-research/policies/journal-policies">self-archiving policies</a>), and these will supersede any other terms that the author or any third party may assert apply to any version of the manuscript.

Acceptance of your manuscript is conditional on agreement, by all authors, with both our <a href="http://www.nature.com/authors/policies/embargo.html">media embargo</a> and <a href="http://www.nature.com/authors/policies/confidentiality.html">confidentiality and pre-publicity</a> policies. In particular, you may arrange your own publicity of the Article (for instance, through your institutional press office), as long as you ensure that journalists strictly adhere to the media embargo.

To assist you in disseminating the work, as soon as the Article is published you will be able to take advantage of the Springer Nature <a href="https://www.springernature.com/gp/researchers/sharedit">SharedIt</a> initiative to <a href="http://authors.springernature.com/share">generate a unique shareable link to the Article</a> that will allow anyone (with or without a subscription) to read it. Recipients of the link who are subscribers will also be able to download and print the PDF.

Thank you for having submitted this work to *Nature Biomedical Engineering*.

Best wishes,

Barbara Cheifet
Editor
Nature Biomedical Engineering

**Open Access** This Peer Review File is licensed under a Creative Commons Attribution 4.0 International License, which permits use, sharing, adaptation, distribution and reproduction in any medium or format, as long as you give appropriate credit to the original author(s) and the source, provide a link to the Creative Commons license, and indicate if changes were made.
In cases where reviewers are anonymous, credit should be given to 'Anonymous Referee' and the source.
The images or other third party material in this Peer Review File are included in the article’s Creative Commons license, unless indicated otherwise in a credit line to the material. If material is not included in the article’s Creative Commons license and your intended use is not permitted by statutory regulation or exceeds the permitted use, you will need to obtain permission directly from the copyright holder.
To view a copy of this license, visit https://creativecommons.org/licenses/by/4.0/
